# High Sensitivity Cardiac Troponin I Detection via MP‐Locked Aptamer and Multimeric DNAzyme‐Coupled Hyperbranched Hybridization Chain Reaction

**DOI:** 10.1002/smll.202512096

**Published:** 2026-02-23

**Authors:** Sayantan Tripathy, Sahil Sharma, Ng Ka Wai, Siddhant Jaitpal, Anthony Dongchau, Saugat Bhattacharyya, Sonya R. Wesselowski, Ashley B. Saunders, Gerard L. Coté, Samuel B. Mabbott

**Affiliations:** ^1^ Department of Biomedical Engineering Texas A&M University College Station Texas USA; ^2^ Center for Remote Health Technologies and Systems Texas A&M University College Station Texas USA; ^3^ School of Computing, Engineering and Intelligent Systems Ulster University Londonderry Northern Ireland UK; ^4^ Department of Small Animal Clinical Sciences College of Veterinary Medicine & Biomedical Sciences Texas A&M University College Station Texas USA; ^5^ Department of Electrical and Computer Engineering Texas A&M University College Station Texas USA

**Keywords:** DNAzyme, hyperbranched hybridization chain reaction, locked aptamer, machine learning, myocardial infarction

## Abstract

Timely and sensitive detection of cardiac troponin I (cTnI) is critical for early diagnosis of myocardial infarction, particularly at the point‐of‐care. Herein, we present a novel colorimetric biosensing platform for high‐sensitivity detection of cardiac troponin I (cTnI). The platform integrates magnetic particle (MP) anchored locked aptamers, stabilized by short complementary strands to minimize nonspecific folding and background activation prior to target binding, with hyperbranched hybridization chain reaction (HCR) and catalytic DNA (DNAzyme) nanocomplex–mediated signal amplification. This enzyme‐free amplification system detects cTnI directly in 25–30 min, with a calculated detection limit of 0.25 ng L^−1^, a wide dynamic range of 0.5–50 000 ng L^−1^, and a coefficient of variation below 5% using just 25 µL of patient serum. The developed assay was evaluated using both human and canine serum samples. To assess classification performance, three distinct hyperparameter optimization strategies were applied to a reduced feature space. The model achieved an accuracy of 90.91% and a recall of 89.89% for human samples, and an accuracy of 83.33% with a recall of 85.71% for canine samples. Blind testing with human serum samples further confirmed the robustness of the platform, showing an overall accuracy of around 90%. This integrated biosensing and machine learning framework enables rapid and sensitive detection of cardiac troponin I, demonstrating strong potential for myocardial infarction diagnosis across species in a pre‐clinical setting.

## Graphical abstract

We report a magnetic particle anchored locked aptamer platform integrated with hyperbranched HCR and DNAzyme amplification for enzyme‐free, ultrasensitive detection of cardiac troponin I. The assay achieves a 0.25 ng/L detection limit within 25 minutes and enables cross‐species MI detection, with machine learning classification reaching 90% accuracy.

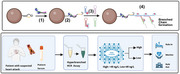

## 1 Introduction

A myocardial infarction (MI), commonly referred to as a heart attack, results from a significant reduction or complete blockage of blood flow to a portion of the heart muscle [1, 2]. According to the 2023 report published by the American Heart Association, an estimated 805 000 heart attacks occur annually in the United States, of which 605 000 are first‐time events, and 200 000 are recurrent [3]. This translates to nearly one heart attack every 40 s, and tragically, around 12% of these cases result in death [4]. Timely diagnosis and intervention remain critical to improving survival rates and patient outcomes. Currently, electrocardiography (ECG) serves as the initial diagnostic tool for MI, particularly effective in identifying ST‐segment elevation myocardial infarction (STEMI) when characteristic changes are present [5, 6]. However, ECG has limited sensitivity, especially in detecting non‐ST elevation myocardial infarction (NSTEMI), where blood tests play a pivotal role in identifying myocardial injury [7, 8].

Biochemical markers, especially cardiac troponins I and T (cTnI and cTnT), are central to the laboratory‐based confirmation of MI [9, 10, 11]. Among these, cTnI is considered the most specific and clinically informative biomarker for myocardial injury due to its distinct release kinetics and higher specificity for cardiac tissue [12]. Clinical guidelines, including the fourth universal definition of MI, emphasize the importance of detecting even small elevations in cTnI for early diagnosis [13]. High‐sensitivity immunoassays performed on automated laboratory platforms such as Siemens Vista 500 [14], ADVIA Centaur XPT [15], and PATHFAST [16] have enabled detection of cTnI concentrations as low as 5–20 ng L^−1^, supporting diagnosis within the first hour of symptom onset [17]. However, these systems typically require large, complex instruments and multistep workflows with high maintenance and operational costs, limiting their use to only centralized laboratory settings. As a result, even when patients with suspected MI reach a hospital within the symptom starting point, the standard diagnostic workflow remains constrained by logistical and infrastructural limitations. The multistep process typically involves tests ordered by a healthcare provider, followed by sample collection and transport to a central laboratory. Then the assay is processed and eventual result reported to the health care provider [18]. This process often incurs turnaround times of several hours. Moreover, patients presenting with intermediate cTnI levels that are near the diagnostic threshold require serial testing at 2–3 h intervals to monitor biomarker trends [19]. Furthermore, the dynamic range of benchtop instruments is often insufficient to cover the entire spectrum of clinically relevant cTnI concentrations (Table 1), particularly for monitoring progression or evaluating borderline cases [19]. These limitations highlight the need for compact, scalable, and high‐dynamic‐range detection platforms suitable for both point‐of‐care (POC) and decentralized clinical use. To address these challenges, POC diagnostics for MI have gained attention for their potential to decentralize testing and expedite clinical decision‐making across diverse settings, including rural clinics, ambulances, and emergency rooms [20]. However, most available POC platforms for cTnI still fall short of the clinical sensitivity required for early detection due to the low abundance of the biomarker during the initial stages of MI [19].

**TABLE 1:** Summary of advancement in cTnI detection methods (commercial and research).

Name of the instrument/Type of work	Limit of Detection (ng L^−1^)	Dynamic Range (ng L^−1^)	Assay time (min)	Downstream Readout	Instrument Requirement	CV at 99th %	Sample volume (approx) (µL)	Refs.
Abbott (i‐STAT)	20	20–50 000	10	Electrochemistry	Yes	16	20	[38]
Philips (Minicare I‐20)	17	18–7000	7	Optical	Yes	19	30	[39, 40]
BioMerieux (Mini Vidas)	19	10‐30 000	20	Fluorescence	Yes, Benchtop	<10	200	[41, 42]
Response Biomedical (RAMP)	30	30–32 000	19	Fluorescence	Yes, Benchtop	20	75	[43, 44]
Siemens (ATELLICA VTLi)	1.24	1.24–1250	8‐20	Optical	Yes	7	100	[45]
LSI Medicine PATHFAST	7	19–50 000	17	Chemiluminescence	Yes, Benchtop	<7	100	[16, 46]
Cortez Diagnostics (RapiCard InstaTest)	1500	1500–5000	5‐15	Colorimetry	No	NR	50	[47]
Nano‐Ditech (Nano‐Check)	100	100–30 000	15	Colorimetry	No	NR	80	[48]
Radiometer AQT90 FLEX TnI	10	10–25 000	20	Fluorescence	Yes, Benchtop	12.9	65	[49]
Lateral Flow immuno assay	10	10–50 000	20	Colorimetry	Yes, Benchtop	5	40	[50]
Aptasensing	0.01 fM	0.1 fM‐1 pM	120	Electrochemiluminesce	Yes, Portable	NR	50	[51]
Lateral Flow assay	0.8	1–10 000	20	Chemiluminescence	Yes, Benchtop	9	50	[52]
Vertical Flow immunoassay	0.2	1–100 000	15	Colorimetry	Yes, Portable	<5	50	[19]
Aptasensing	0.4	0.5–50 000	120	Surface‐enhanced Raman scattering	Yes, Portable	<5	25	[30]
Our work	0.25	0.5–50 000	25	Colorimetry	Yes	<5	25	

To overcome the limitations of commercial high‐sensitivity platforms and underperforming POC devices, researchers have increasingly turned to electrochemical immunosensor approaches as promising alternatives for commercial cTnI detection. Electrochemical immunosensors and microfluidic devices have demonstrated promise in enhancing sensitivity, but issues such as limited shelf‐life, temperature instability, and the requirement for sample preprocessing reduce their practical utility [21, 22, 23, 24]. Paper‐based lateral flow assays (LFAs), though portable and easy to use, often suffer from poor sensitivity and cannot detect cTnI at levels below diagnostic thresholds [25].

As an alternative, aptamer‐based detection systems have emerged due to their high specificity, stability, and synthetic accessibility [26]. Certain cTnI‐specific aptamers, such as Tro4 and Tro6, exhibit stronger binding affinity (270 pM) than conventional antibodies (20 nM) [27]. However, aptamer performance can be compromised under thermal and environmental stress, which often induces partial unfolding or misfolding of the aptamer structure [28]. Such conformational instability can lead to non‐specific binding, reduced target affinity, and elevated background signals, particularly problematic in field‐deployable or POC applications where temperature control is limited [29]. To address this, locked aptamer designs have been developed, incorporating complementary sequences or structural constraints that stabilize the aptamer's native conformation in the absence of the target [30]. Upon target binding, these “locked” regions are displaced, allowing proper folding and high‐affinity interaction. This strategy enhances structural integrity and binding specificity, effectively reducing false positives and improving assay reliability even under fluctuating environmental conditions [31, 32, 33]. However, to achieve the sensitivity necessary for early‐stage detection (<20 ng L^−1^), aptamers often require signal enzymatic PCR based or isothermal amplification strategies, such as nanoparticle or nucleic acid‐based sandwich assays. These systems, while sensitive, involve multistep workflows. Additionally, they require costly and labile reagents, benchtop instruments, and nonspecific amplification making them less suitable for robust POC deployment [23, 34, 35].

Colorimetric biosensing platforms, particularly those utilizing DNAzyme catalysis, offer a powerful and practical alternative for POC diagnostics. Moreover, these platforms are generally more robust under environmental fluctuations, cost‐effective, and compatible with paper‐based and microfluidic systems. However, a key limitation of colorimetric detection is its inherent poor sensitivity [36]. It typically lacks the ability to reliably detect very low analyte concentrations, especially in the sub‐nanogram per liter range, which is often required for early‐stage high‐sensitivity cTnI detection [37]. This limitation has been well documented in literature and presents a challenge for clinical applications where ultrasensitive quantification is critical. Table 1 summarizes the existing commercial kits and emerging approaches from the literature developed for cardiac troponin I detection, highlighting their respective detection mechanisms, sensitivity ranges, and clinical utility.

To address the research gaps, in this study, we present an innovative colorimetric diagnostic platform for high‐sensitivity cTnI detection that addresses both sensitivity and accessibility challenges by integrating magnetic particle‐based locked aptamer sensing with a non‐enzymatic hyperbranched hybridization chain reaction and catalytic DNAzyme‐driven signal amplification. This combined strategy enables highly specific and visually detectable cTnI recognition, offering a robust, instrument‐free solution suitable for rapid, point‐of‐care myocardial infarction diagnosis in resource‐limited settings. MPs functionalized with cTnI‐specific aptamers enable selective and specific cTnI capture and separation from complex biofluids. Upon binding to cTnI, a structural change in the aptamer unlocks a concealed initiator strand that triggers a downstream HCR between partial complementary metastable DNA hairpins [53]. To further enhance signal amplification, we have employed a hyperbranched HCR design, in which a secondary set of hairpins initiates branch extension from the primary HCR product [54]. This leads to a dense DNA nanostructure with significantly increased loading of multimeric catalytic DNAzyme units. This catalytic system forms when a guanine‐rich DNA sequence folds into a triplex/quadruplex structure and binds to hemin, creating a DNAzyme that mimics the peroxidase activity of natural enzymes like horseradish peroxidase [55]. In the presence of hydrogen peroxide (H_2_O_2_), the DNAzyme catalyzes the oxidation of chromogenic substrates, generating a visible color change typically blue or green which can be seen by the naked eye or quantified using simple image analysis. This dual‐layered amplification through hyperbranched HCR and hemin‐incorporated DNAzyme produces a robust colorimetric readout. As noted in Table 1, our assay achieved a calculated detection limit of 0.25 ng L^−1^ for cardiac troponin I, well below the clinical threshold for early myocardial infarction, with a dynamic range of 0.5 to 50 000 ng L^−1^, turnaround time of 25 min, and volume of 25 µL, which is suitable for rapid diagnostics in critical care settings. The assay was validated on 22 human and 18 canine serum samples, with the high (∼99% CV of <5) troponin I sequence homology supporting its translational potential across species.

## 2 Results and Discussion

### 2.1 Principle of the Assay

**FIGURE 1:**
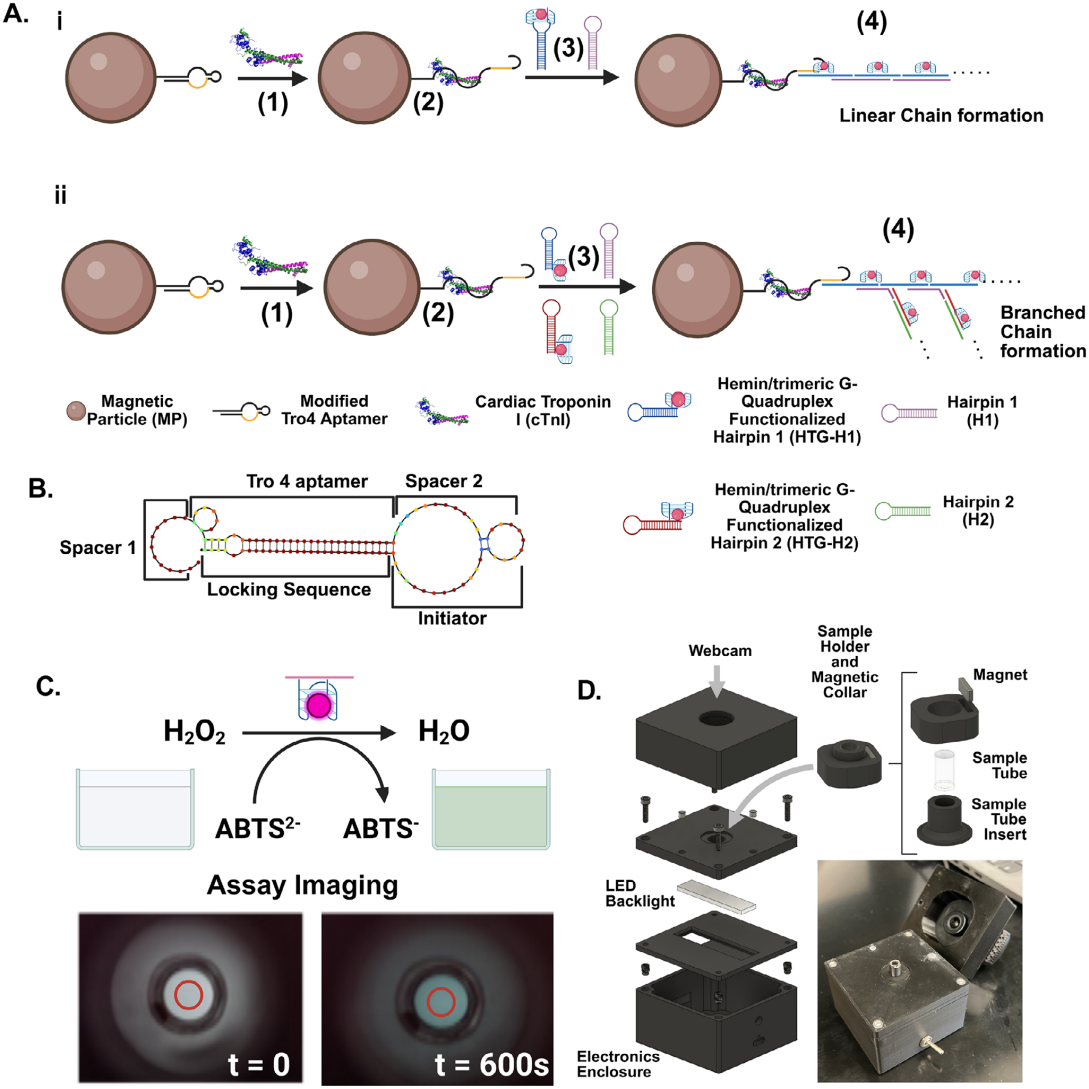
Schematic representation of the colorimetric assay for cTnI detection using a DNAzyme‐coupled hybridization chain reaction platform and integrated imaging system. (A) Stepwise mechanism of the MP‐based detection assay with linear and hyperbranched chain reaction. (1) Streptavidin‐coated MPs are functionalized with biotinylated locked Tro4 aptamer, which specifically binds to cTnI. (2) Upon incubation with the sample, cTnI binds to the immobilized aptamer, results the unfolding of locked structure. (3) The cTnI–aptamer complex triggers hybridization chain reaction (HCR) through sequential binding of four partial complementary DNA hairpins, modified with hemin coupled trimeric triplex and G quadrupex DNAzyme motif. (4) The growing HCR amplicon amplifies the signal with DNAzyme units that exhibit peroxidase‐like catalytic activity. A (i), shows the linear chain amplification using two hairpins. (A(ii)) shows the hyperbranched chain amplification using four hairpins. (B) Probable structure and functional part of the engineered aptamer. (C) Principle of colorimetric detection. The DNAzyme catalyzes the oxidation of ABTS^2^^−^ to ABTS^.^^−^ in the presence of hydrogen peroxide (H_2_O_2_), leading to a visible color change from colorless to green. The bottom panel shows representative assay images captured at 0 and 600 s, illustrating the time‐dependent color development due to the DNAzyme activity. (D) Schematic and physical setup of the custom‐built imaging platform used for real‐time assay monitoring. The setup includes a sample holder with an integrated magnetic collar to immobilize magnetic particles during the reaction, a webcam for image acquisition, and an LED backlight for consistent illumination. The exploded view shows the layered construction of the enclosure containing the sample tube insert, electronics, and camera alignment components. The assembled system enables low‐cost and portable imaging for colorimetric quantification.

The assay employs the conformational switching behavior of a locked aptamer that we have previously validated to ensure specific recognition of cTnI in complex biofluids [30, 56]. The aptamer is conjugated to MPs along with a sequestered HCR initiator. In the presence of cTnI, the aptamer unfolds, releasing the initiator strand that triggers signal amplification. The modified Tro4 aptamer shown in Figure 1B is annotated to indicate its functional domains. Spacer 1 consists of up to 12 nucleotides and includes a biotin modification at the 5′ end for attachment to MPs, providing structural flexibility. The aptamer region, which specifically binds to cTnI, is stabilized by a complementary locking sequence. A second spacer and an initiator sequence are positioned to form a loop that connects the aptamer to the locking region, enabling controlled activation for downstream signal amplification. The thermodynamic profile of the engineered aptamer is described in Figure S1. First, we optimized our assay with a linear HCR reaction where amplicons grow at single direction with two hairpins, which can be called as first generation or linear hybridization chain reaction (Figure 1A(i)). In this assay, the magnetic particle‐bound locked aptamer coupled with the HCR initiator opens up in presence of cTnI. The unfolded HCR initiator initiates a cascade reaction wherein two partially complementary DNAzyme‐containing hairpins undergo hybridization, leading to the formation of a linear chain. The resulting amplicons from the hybridization chain reaction were subsequently incubated with hemin to form a stable triplex structure. This triplex structure exhibits horseradish peroxidase‐like activity in the presence of hydrogen peroxide and 2,2'‐azinobis‐(3‐ethylbenzothiazoline‐6‐sulfonic acid) (ABTS). The intensity of color is directly proportional to the amount of cTnI present (Figure 1C). To ensure consistent and efficient catalytic activity in the final colorimetric reaction, hemin was pre‐incorporated into the hairpin sequences. This integration eliminates the need for a separate hemin addition step, thereby reducing the number of assay steps and significantly shortening the overall assay time. Additionally, we optimized the amplification architecture by modifying Hairpin 1 to include an initiator sequence at its 3′ end. This initiator is capable of triggering the hybridization of additional hairpins, thereby initiating secondary amplification cascades. This design enables a transition from a first‐generation linear HCR which forms a single, unbranched polymer chain, to a hyperbranched HCR, characterized by multiple initiation points and exponential signal amplification. As illustrated in Figure 1A(ii), this hyperbranched architecture leads to faster and more intense signal generation, ultimately improving the sensitivity and detection speed of the assay.

To facilitate the colorimetry, we built a small, low‐cost imaging system, as shown in Figure 1D. The assay is conducted in a small cylindrical flat‐bottomed sample tube, which is inserted into a 3D‐printed holder. We also designed a collar embedded with a small, rectangular N52 magnet that can be slipped over the neck of the holder to magnetically separate the MPs. The holder is mounted onto a sample plate with an LED backlight positioned beneath it to transmit light through the sample tube. A simple circuit, consisting of a switch, rechargeable battery, and variable resistor for adjusting the LED backlight's luminosity, is housed within an electronics enclosure. Images of the backlit sample are captured using a manual focus webcam mounted in a 3D‐printed lid, with the aperture suspended directly above the sample tube. Alignment and blocking of external light are ensured by magnetically coupling the lid to the sample plate. Images are collected from the assay at user‐specified intervals (usually every 30 s) using a custom Python program operated via a graphical user interface.

### 2.2 Oligonucleotide Screening NUPACK

To evaluate the feasibility of implementing a hyperbranched hybridization chain reaction for signal amplification, we conducted a systematic in silico design and screening of hairpin sequences using NUPACK. The hairpins were designed with partial sequence complementarity to enable controlled strand displacement and facilitate progressive branch formation. To further enhance amplification efficiency, an additional initiator sequence was appended to the 3′ end of hairpin H1, allowing a secondary set of hairpins with DNAzyme [57] to hybridize and propagate the reaction in multiple directions, thus generating a hyperbranched architecture. Over 100 candidate designs were computationally assessed for their ability to support hierarchical and sequential hybridization required for hyperbranched propagation, while minimizing undesired secondary structures and off‐target interactions. This simulation‐driven approach enabled us to pre‐select sequences that remain in a stable, closed conformation in the absence of both the modified Tro4 aptamer and the target protein, cTnI, thereby reducing the risk of spontaneous hybridization and background signal. While similar computational strategies have been useful in prior work [30], this design was tailored to support the unique structural complexity and amplification dynamics of a hyperbranched HCR system, ensuring both reliability and cost‐efficiency in probe development. The simulation allowed us to rank the hairpin candidates based on the predicted thermodynamic stability of undesired secondary structures and cross‐hybridization products under the experimental conditions (25°C, 1000 nM concentration of each strand). From this screening, four hairpins HTG‐H1, H1, HTG‐H2, and H2 were selected as optimal components for the hyperbranched HCR system. Figure S2C summarizes the predicted structures and concentrations of the major species formed when these hairpins are combined in silico at 25 °C. The NUPACK output revealed that, aside from the intact hairpins, only three minor side products, labeled as (2), (3), and (4), are formed in small but detectable concentrations. Importantly, HTG‐H1, the hairpin designed to bind specifically to the initiator strand exposed only upon conformational switching of the modified aptamer upon cTnI binding, remained entirely in its closed state at a concentration of 1000 nM in the simulation (species (1)). This indicates strong structural fidelity and resistance to unintended activation in the absence of the analyte (Figure S2). This finding is critical for the performance of the assay: since the initiator strand is only revealed when the aptamer binds to its target (cTnI), HTG‐H1's inactivity in its absence ensures that the hyperbranched HCR does not nonspecifically self‐assemble. Consequently, any untriggered hairpin complexes that remain can be efficiently removed through washing steps, significantly reducing the non‐specific background signal in the downstream colorimetric detection. This robust sequence‐level control over unwanted activation is a cornerstone of our assay's specificity and high signal‐to‐noise ratio.

### 2.3 Validation of NUPACK Results by Gel Electrophoresis and Atomic Force Microscopy (AFM)

**FIGURE 2:**
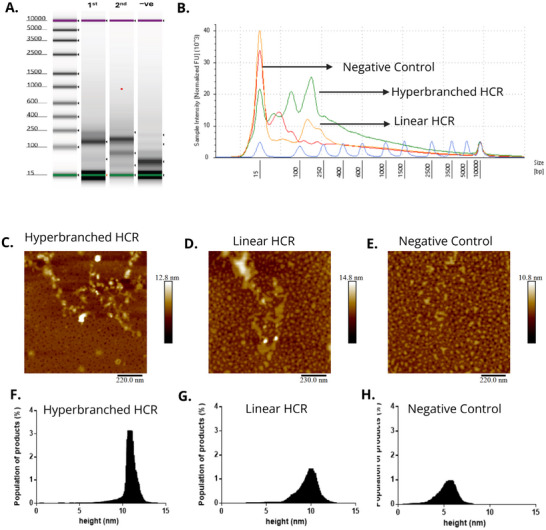
Characterization of linear and hyperbranched HCR products using electrophoresis, AFM, and height profiling. (A) gel showing HCR amplification under three conditions: linear HCR (locked aptamer, 50 ng L^−1^ cTnI, HTG‐H, H1), hyperbranched HCR (locked aptamer, 50 ng L^−1^ cTnI, HTG‐H1, H1, HTG‐H2, H2), and negative control (locked aptamer, HTG‐H1, H1, HTG‐H2, H2, no cTnI). Hyperbranched HCR displays higher molecular weight bands, indicating extensive amplification, while the negative control shows minimal product. (B) Electropherogram comparing product size distributions. Hyperbranched HCR (green) exhibits broad, high‐intensity peaks spanning larger base pair regions than linear HCR (orange), confirming multivalent amplification. The negative control (blue) shows weak background signals. Hyperbranched HCR (C) shows dense, branched networks; linear HCR (D) shows elongated, linear structures; and the negative control (E) shows minimal structural features. Hyperbranched HCR (F) shows the tallest structures (10–12 nm), followed by linear HCR(G) (7–9 nm). The negative control (H) shows low, non‐specific features (<5 nm), consistent with unassembled components. These results confirm successful target‐induced HCR and demonstrate that hyperbranched architecture yields more complex and higher‐mass DNA structures compared to linear HCR.

To confirm the formation and structural complexity of the linear and hyperbranched HCR products, we employed multiple complementary characterization techniques, including gel electrophoresis via Agilent TapeStation analysis, AFM, and height distribution profiling from AFM topography data. Figure 2A shows the electrophoretic profiles of the HCR products obtained under three experimental conditions: (i) Linear HCR, using locked aptamer, 50 ng L^−1^ of cTnI, and HTG‐H1 and H1 (denoted as first in the Figure 2A); (ii) Hyperbranched HCR, with the same target and aptamer but using four hairpins (HTG‐H1, H1, HTG‐H2, and H2) (denoted as second in the Figure 2A); and (iii) Negative control, which includes locked aptamer HTG‐H1, H1, HTG‐H2, and H2, but no cTnI target control denoted as −ve in the Figure 2A). The linear HCR product showed a distinct band shift to a higher molecular weight compared to unreacted hairpins, while the hyperbranched condition displayed a broader, more diffuse smear extending further upward, indicates of increased molecular weight and polymer complexity. In contrast, the negative control showed only very faint or unshifted bands near the hairpin baseline, confirming that target‐induced aptamer switching is essential for initiating HCR amplification. The resulting HCR chains are nicked and linear, which lacks defined secondary or tertiary structures. Due to this reason, their electrophoretic migration does not follow conventional size‐based proper banding, making it a little difficult to directly interpret structural features from the gel image alone. Therefore, we further analyzed the TapeStation output using electropherogram profiles, which allowed better visualization and differentiation of the molecular weight distribution across the three experimental conditions. In Figure 2B, the corresponding electropherogram profiles further illustrate the distribution of amplicons across different base pair lengths. The hyperbranched HCR (green curve) exhibits a broader and more intense set of peaks extending to higher molecular sizes above 1000 bp, confirming the formation of large, branched DNA assemblies. In contrast, the first‐generation linear HCR (orange curve) shows a narrower size distribution primarily below 500 bp. The negative control (blue curve) displays a low background signal with minor peaks, representing nonspecific hybridization or unreacted hairpins. This comparative analysis also validates that the inclusion of additional hairpins in the hyperbranched scheme significantly enhances the amplification potential and structural complexity of DNA assemblies.

AFM imaging was performed to visualize the nanostructures formed under the same three experimental conditions. Figure 2C shows AFM topography of the hyperbranched HCR products, which appear as dense, irregular, and interconnected DNA networks indicative of extensive amplification and branched assembly. Figure 2D, representing the linear HCR, shows relatively linear, thread‐like features of moderate length, consistent with the unidirectional assembly of DNA polymers from two hairpins [58]. Figure 2E, corresponding to the negative control, exhibits no defined structures, further validating that target‐induced structural switching of the locked aptamer is essential for HCR initiation. AFM height distribution analysis provides additional evidence of structural amplification. The height histogram in Figure 2F shows that hyperbranched HCR products predominantly exhibit heights around 10–12 nm, significantly higher than the linear HCR products in Figure 2G, which are centered around 7–9 nm. In contrast, the negative control shows only background signals and scattered particles with heights less than 5 nm, likely corresponding to unhybridized oligonucleotides or nonspecific aggregates (Figure 2H). The progressive increase in average height from negative control to linear HCR, and from linear to hyperbranched HCR, directly correlates with the increasing complexity and size of the DNA nanostructures formed. These results collectively confirm the successful formation of branched DNA assemblies in the presence of target cTnI and validate the effectiveness of the locked aptamer as a switchable initiator for target‐specific HCR amplification. Notably, the total area occupied by the hyperbranched HCR structures is significantly higher than that of linear HCR, despite a lower population count, underscoring the amplification efficiency and spatial expansion of the hyperbranched mechanism (Figure S3). This area‐to‐population shift is consistent with the increased height distribution previously observed. The data in Figure S3 also provide complementary morphological evidence of the progressive structural complexity of HCR products, further validating the formation of high‐density branched DNA networks in response to cTnI‐triggered aptamer opening.

### 2.4 Optimization of the Stability of Color Using G‐Quadruplex and ‐Trimeric Triplex

**FIGURE 3:**
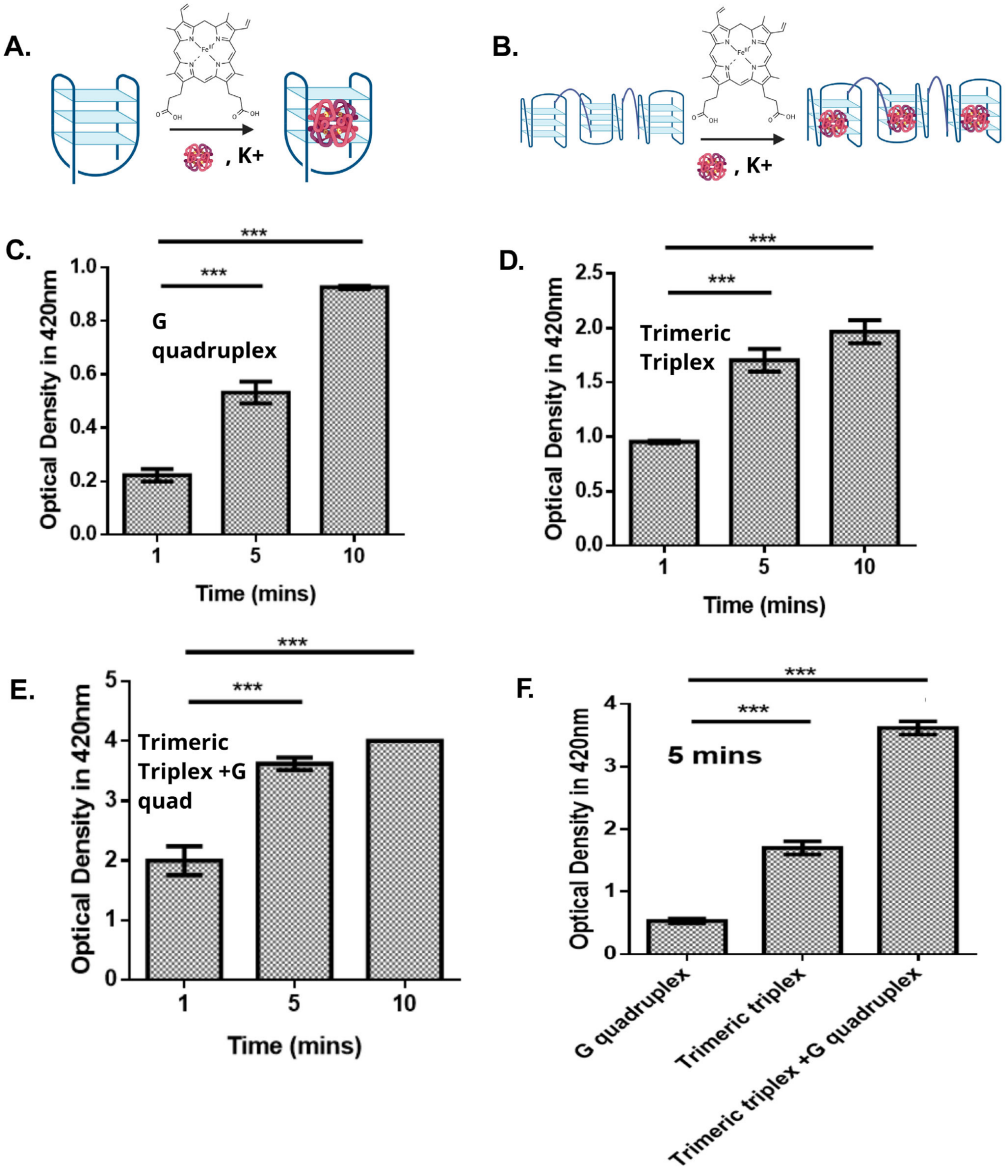
Schematic representations of ABTS^2^^+^ generation using (A) G‐quadruplex and (B) trimeric triplex DNA structures in the presence of K^+^ and hemin. Both structures catalyze the oxidation of ABTS by H_2_O_2_ to produce the green‐colored radical cation ABTS^2^^+^, detectable at 420 nm. Time‐dependent optical density measurements at 420 nm for ABTS^2^^+^ generation using (C) G‐quadruplex, (D) trimeric triplex, and (E) a mixture of trimeric triplex and G‐quadruplex over 1, 5, and 10 min. While G‐quadruplex shows moderate activity, the trimeric triplex generates significantly stronger signals, and the mixture of both shows the highest catalytic output, reaching saturation within 5 min. (F) Comparative analysis at the 5‐minute mark reveals that the mixed system produces a significantly higher signal than either structure alone. All data represent mean ± SD (*n* ≥3), and statistical significance was assessed using one‐way ANOVA (***p < 0.001).

Following the structural characterization of linear and hyperbranched HCR assemblies, we next investigated their catalytic performance in colorimetric signal generation. Specifically, we evaluated the efficiency and stability of two DNAzyme motifs, the conventional G‐quadruplex and a multimeric trimeric triplex DNAzyme, when incorporated into the HCR system. Prior to assessing their peroxidase‐mimicking activity, we first optimized the potassium ion concentration in the colorimetric buffer to ensure optimal G‐quadruplex formation, which is known to be highly dependent on the presence of K^+^. The G‐quadruplex structure, formed by guanine‐rich DNA sequences in the presence of monovalent cations such as K^+^, provides a stable scaffold for binding hemin, which is a prosthetic group derived from hemoglobin. When hemin intercalates into the planar G‐quartet of the folded G‐quadruplex, it mimics the catalytic environment of natural peroxidase enzymes. This G‐quadruplex hemin DNAzyme complex exhibits robust peroxidase‐like activity, catalyzing redox reactions in the presence of hydrogen peroxide (Figure 3A,B). We found that 20 mM KCl was the optimal concentration for maximizing G‐quadruplex‐mediated color generation (Figure S4A). While both Na^+^ and K^+^ can facilitate G‐quadruplex folding, their effects are kinetically and thermodynamically distinct. Na^+^ stabilizes G‐quadruplex structures with relatively low affinity and slower folding kinetics, typically favoring antiparallel conformations that exhibit reduced catalytic efficiency and delayed color development. In contrast, K^+^ promotes rapid formation of parallel or hybrid G‐quadruplex topologies with high affinity, providing an optimal scaffold for hemin binding and efficient peroxidase‐like activity [59, 60]. Consistent with this behavior, we observed minimal and slowly developing color in Na^+^ alone, whereas the presence of K^+^ resulted in rapid and intense signal generation. Importantly, Na^+^ concentrations did not diminish catalytic activity when K^+^ was present, indicating that K^+^ dominates G‐quadruplex folding and stabilizes the catalytically active conformation (Figure S4B).

To further confirm that the G‐quadruplex motif within the HCR hairpins folds into a catalytically active conformation under these conditions, HTG‐H2 was pre‐incubated as a representative hairpin with hemin prior to activity measurements, as hemin coordination is dependent on proper G‐quadruplex folding and can be kinetically limiting. In the presence of 20 mM K^+^ ions, pre‐incubated HTG‐H2 exhibited a clear concentration‐dependent increase in ABTS radical production upon addition of H_2_O_2_which confirms peroxidase activity. In contrast, negligible color development was observed in the absence of either K^+^ or hemin. These results provide direct functional evidence that the G‐quadruplex within the HCR hairpins adopts its catalytically active conformation under the optimized assay conditions (Figure S4C). Given that hemin was introduced from a citrate–phosphate buffer to enable DNAzyme formation, we next evaluated whether this step affected DNA structural integrity. Hemin was prepared in citrate–phosphate buffer (pH 5.5) and added to pre‐folded HTG H1 hairpins maintained in the colorimetric buffer. To assess stability, we compared Hemin‐incorporated HTG H1 (exposed to citrate–phosphate buffer during hemin addition) with native HTG H1 prepared in the absence of hemin and without exposure to citrate–phosphate buffer. Native PAGE analysis revealed no detectable DNA degradation or structural alteration in either condition, confirming that transient exposure to citrate–phosphate buffer does not compromise DNA integrity or G‐quadruplex formation. These results demonstrate that the DNAzyme remains structurally stable and fully functional throughout the colorimetric reaction (Figure S4D). Higher concentrations of potassium led to nonspecific signal development, indicating the potential for false positives; thus, precise potassium tuning was essential. However, G‐quadruplex‐based DNAzymes are limited by relatively low catalytic efficiency and poor signal stability. The color generated often fades within minutes, making them unsuitable for long‐term kinetic monitoring. Additionally, the intensity of the color is modest, which can reduce assay sensitivity, particularly in low target abundance scenarios. To overcome these limitations, we designed a multimeric DNAzyme based on a trimeric triplex motif capable of binding multiple hemin units. This configuration promotes stronger and more sustained peroxidase‐like activity through multivalent hemin coordination and improved structural rigidity. We compared the performance of this trimeric triplex DNAzyme with that of the conventional G‐quadruplex by incorporating each structure into the hairpin 1 (HTG‐H1) and evaluating colorimetric output under identical reaction conditions in the presence of 20 mM potassium ions. The trimeric triplex‐modified H1 generated significantly more intense and stable color, which persisted over an extended period, enabling robust real‐time signal tracking and enhanced signal‐to‐background discrimination. To further assess the catalytic contribution of each motif in the context of the hyperbranched HCR system, we compared three conditions: (i) HCR with only G‐quadruplex‐modified strands, (ii) HCR with only trimeric triplex‐modified strands, and (iii) HCR with a mixture of both, where one set of hairpins carried the G‐quadruplex motif and the other carried the trimeric triplex. This comparison allowed us to evaluate the additive or synergistic effects of combining both catalytic architectures within the amplification cascade. The peroxidase‐mimicking activity of these DNA structures was assessed using the ABTS/H_2_O_2_ system, and optical density was monitored at 420 nm at different time points (1, 5, and 10 min). As shown in Figure 3C, the G‐quadruplex alone demonstrated a time‐dependent increase in ABTS^2^^+^ production, with a gradual enhancement in optical density from 1 min to 10 min. However, the signal intensity remained relatively modest, indicating a lower catalytic efficiency compared to other architectures. This slower signal development suggests that while the G4‐hemin complex forms and is catalytically active, the structural conformation or hemin accessibility may limit its maximal activity under the given conditions.

In contrast, the trimeric triplex structure exhibited a substantially enhanced catalytic output (Figure 3D). The ABTS^2^^+^ signal increased steeply and nearly saturated within 10 min, with optical density values significantly higher than the G quadruplex group at all time points. The triplex‐hemin complex appears to offer improved hemin stabilization or enhanced substrate accessibility, resulting in a faster and stronger colorimetric response. Strikingly, the combination of the trimeric triplex and G‐quadruplex motifs produced a highly intense color signal that saturated as early as 5 min (Figure 3E). The synergistic contribution of both DNAzymes within this mixed architecture appears to dramatically accelerate the ABTS oxidation process, with the optical density at 5 min surpassing even the 10‐minute signals observed for either component alone. This is further evident in the comparative analysis at the 5‐minute mark (Figure 3F), where the mixed system (trimeric triplex + G‐quadruplex) significantly outperformed both individual motifs (*p* < 0.001). These findings highlight the enhanced catalytic capability of the hybrid system, likely due to cooperative effects between the two DNA motifs. The structural multiplicity in this system may facilitate multiple hemin binding sites or more efficient substrate channeling, leading to rapid accumulation of ABTS^2^^+^. However, when implementing this system in a hyperbranched hybridization chain reaction, we observed that incorporating trimeric triplex structures in all hairpins resulted in overly dense and rapid color generation (Figure S5). This excessive signal intensity can mask concentration‐dependent differences and may lead to false‐negative interpretations due to signal oversaturation. To address this, we strategically incorporated the trimeric triplex motif in only one set of hairpins while using the G‐quadruplex motif in the other set. This configuration provided better dynamic range, more interpretable signal intensities, and maintained the strong colorimetric response within an optimal reaction window.

### 2.5 Determination of the Assay Sensitivity

**FIGURE 4:**
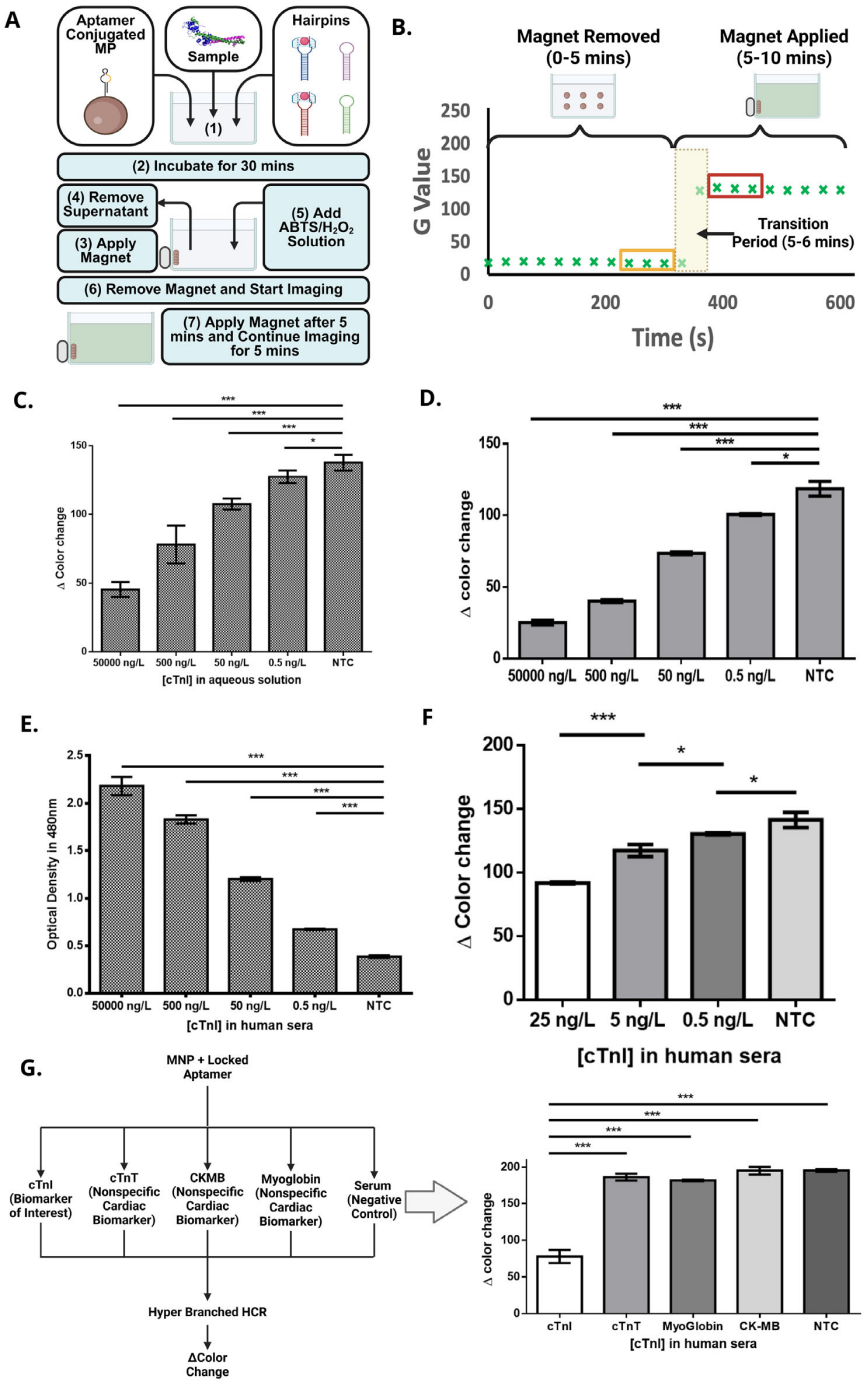
Workflow, kinetic response, and analytical validation of the colorimetric cTnI assay. (A) Schematic of the assay protocol: (1) Aptamer‐conjugated MPs are incubated with cTnI and HCR hairpins (20 min). (2) Complexes are magnetically separated and washed. (3) ABTS/H_2_O_2_ is added for color development. (4) Magnet is removed for imaging (0–5 min). (5) Magnet reapplied for enhanced signal capture (5–10 min). (B) Real‐time kinetic profile showing low G values during 0–5 min (magnet removed) and increased signal upon reapplication (5–10 min), with a clear transition at ∼300–360 s. (C) ΔG values for cTnI standards (50 000–0.5 ng L^−1^) and NTC in aqueous solution (*n* = 3) (^***^ *p* < 0.001). (D) ΔG values for cTnI in spiked human serum (50 000–0.5 ng L^−1^), showing dose‐dependent response (^*^ *p* < 0.05). (E) Spectrophotometric validation (480 nm absorbance) in spiked serum, consistent with image analysis (*n* = 3) (^*^ *p* < 0.05, ^***^ *p* < 0.001). (F) Resolution across human serum replicates (25–0.5 ng L^−1^), showing good reproducibility in healthy controls with expected variability in NTC (*n* = 3) (^*^ *p* < 0.05) (^***^ *p* < 0.001). (G) Specificity Test of the assay with other closely related biomarkers such as cTnT, CK‐MB and Myoglobin (^***^ *p* < 0.001).

After confirming the proof of concept for signal amplification and color generation in the presence of the target, first, we evaluated the assay's sensitivity using synthetic cTnI. As previously described, the assay was conducted in a small, cylindrical, flat‐bottomed sample tube placed within a 3D‐printed holder housed inside a custom blackbox color analyzer. A manually focused webcam, mounted on a 3D‐printed lid and aligned directly above the sample tube, ensured consistent optical conditions. A lid was placed over the sample to minimize ambient light interference. Images were captured every 30 s using a custom Python script, which extracted average RGB values from ∼300 central pixels per frame. The hairpins used in this HCR system contained an integrated trimeric triplex DNAzyme that catalyzes ABTS oxidation in the presence of H_2_O_2_, producing a green ABTS^2^^+^ signal visible to the eye and quantifiable by imaging or spectrophotometry. While the linear HCR assay achieved a low detection limit of 0.05 ng L^−1^ in buffer (Figure S6), its sensitivity dropped significantly in human serum, with a detection limit of 500 ng L^−1^ (Figure S7), well above the clinical threshold (50 ng L^−1^). This reduced performance was likely due to matrix effects that hinder hybridization and signal generation. Attempts to improve signal by extending ABTS reaction time and using magnetic separation were also unsuccessful in serum, highlighting the need for a more robust amplification strategy prompting the development of the hyperbranched HCR system. This approach allows sufficient time for multiple DNAzyme structures to generate multiple branched amplicons, which in turn interact with the ABTS substrate in the presence of hydrogen peroxide, thereby maximizing signal output through their peroxidase‐mimicking activity. Based on prior validation, we designed the HCR hairpins with a trimeric triplex DNAzyme incorporated into the H1 strand (HTG‐H1) and a G‐quadruplex motif into the H2 strand (HTG‐H2), creating a synergistic catalytic system. This hyperbranched architecture significantly improved analytical sensitivity, enabling detection below 0.5 ng/L of target analyte. However, at higher analyte concentrations, the resulting color signal became overly intense, leading to poor quantifiable differentiation and risk of false interpretation due to oversaturation (Figure S8). To address this challenge, we introduced a magnetic separation step about 4 min after initiating the DNAzyme‐catalyzed ABTS/H_2_O_2_ reaction. This intervention effectively stopped further color development and stabilized the signal for consistent optical analysis. With this modified protocol, we observed clear and distinguishable color differences across a wide concentration range, as confirmed through analysis of red, green, and blue (RGB) channel intensities (Figure S9). This strategy ensured accurate quantification while preserving the rapid, high‐sensitivity advantages of the hyperbranched HCR system. Figure 4A outlines the workflow of the assay. At first, samples containing cTnI are incubated with aptamer‐functionalized MPs and hairpin DNA probes. After a 20‐minute incubation period (maximum 30 minutes to avoid non specific HCR), a magnet is used to immobilize the MPs and separate them from unreacted reagents. The ABTS/H_2_O_2_ substrate is then added, and color development is monitored using a time‐segmented imaging strategy and MPs were subsequently removed to reduce background noise and ensure a consistent optical path for RGB quantification. This stepwise workflow allowed for controlled, reproducible signal generation and quantitative evaluation of cTnI levels across different sample types. Figure 4B illustrates how the imaging process is temporally controlled for optimal signal capture. In the first 5 min (1‐minute ABTS background + 4‐minute H_2_O_2_ reaction), images are acquired without a magnet to establish a baseline signal, followed by 5 min with a magnet applied to localize the MP‐bound complexes and intensify the color signal through concentration of the DNAzyme‐rich HCR products. To quantify the colorimetric signal generated by the DNAzyme‐catalyzed ABTS reaction, we first converted the time‐lapse images of the reaction wells into grayscale using image analysis software. In this format, each pixel is assigned a brightness value, darker pixels correspond to stronger green coloration from oxidized ABTS, allowing us to measure the intensity of color development over time in a simplified and consistent manner. For each concentration, the grayscale intensity was measured at each time frame across the duration of the reaction. The delta intensity values (Δ) were then calculated by subtracting the grayscale value at the baseline (initial frame, prior to color development, 3 points) from the value at the endpoint (final frame, after color saturation, 3 points) (Figure S10). This approach provides a consistent metric for comparing signal generation across concentrations by capturing the net change in intensity due to the reaction. The grayscale‐based method enables objective, reproducible quantification of colorimetric output, which is essential for comparing performance across different experimental conditions. A distinct increase in delta value (greyscale) is observed following magnet application, with the transition period between 300–360 s marking the onset of significant color development due to localized catalytic activity. Notably, the initial signal is already relatively dark due to the inherent black color of the magnetic particles, which contributes to a low baseline grayscale intensity. At high cTnI concentrations, the colorimetric product becomes deeply saturated, further darkening the final signal. Because both the starting and final signals are dark, the change in grayscale (Δ) appears smaller when higher cTnI is present. In contrast, lower cTnI concentrations result in less saturated color, allowing for a more noticeable shift from the dark baseline, thus yielding a higher shift in Δ. Notably, the baseline grayscale intensity following magnet application is inherently low due to the intrinsic dark color of the magnetic particles. As cTnI concentration increases, enhanced HCR amplification produces greater DNAzyme loading and deeper ABTS^2^^+^ coloration, resulting in darker endpoint images. Because the grayscale metric is calculated as a baseline‐normalized difference (Δ), higher absolute signal intensities may yield smaller Δ values when both baseline and endpoint signals are dark. This effect reflects the nature of differential image analysis rather than signal saturation or loss of analytical dynamic range. The quantitative performance of the hyperbranched assay was first assessed in aqueous buffer conditions (Figure 4C). As predicted a clear, inverse correlation of color change from baseline between high and low cTnI concentration was observed. Even at low concentrations such as 0.5 ng L^−1^, a significant change in signal was observed compared to the NTC, which exhibited the least color due to no HCR in the absence of target. When the assay was applied to human serum samples (Figure 4D), the same trend was preserved, with slightly higher signal intensities likely due to matrix effects such as serum protein interference and viscosity which sometimes causes nonspecific opening of hairpins. Nonetheless, the system retained clear differentiation across clinically relevant cTnI concentrations, affirming its suitability for biological sample analysis.

To further corroborate the colorimetric readout, absorbance at 480 nm corresponding to ABTS^2^^+^ was measured in serum samples (Figure 4E). Importantly, absorbance measurements at 480 nm confirm a monotonic increase in signal with increasing cTnI concentration, demonstrating that the colorimetric response does not saturate within the tested range. These results support the digital imaging data; higher cTnI concentrations yielded significantly higher absorbance values, consistent with high DNAzyme generation via increased HCR. The NTC again displayed minimal absorbance. Finally, the resolution and reliability of the assay were evaluated through coefficient variation analysis across biological replicates (Figure 4F). While CVs increased slightly at lower cTnI concentrations (normal range), they remained within acceptable analytical margins, demonstrating good reproducibility even at the sub‐picogram level (less than 5%). The NTC, consistent with its high background signal and stochastic amplification, exhibited the highest CV, indicating more variability in nonspecific HCR events. Following that, we performed specificity testing using structurally related cardiac biomarkers, including CK‐MB, cTnT, and myoglobin, all of which showed negligible cross‐reactivity with our assay, confirming its high target specificity (Figure 4G). Under identical assay conditions, cTnI produced a significantly lower color‐change compared to all non‐target proteins and the no‐target control (NTC). Statistical analysis (one‐way ANOVA) revealed that the cTnI signal was significantly distinct from cTnT, CK‐MB, myoglobin, and the NTC (*p* < 0.001), while no statistically significant differences were observed among the non‐target proteins and the NTC. These results indicate minimal cross‐reactivity and confirm that HCR initiation and DNAzyme amplification are highly specific to cTnI binding. Following validation of the assay's specificity and sensitivity, we extended our testing to real clinical samples to further assess its performance. We tested 22 human serum samples and, for comparative analysis, included 18 canine serum samples. While our primary goal is the detection of human cTnI for clinical diagnostics, canine cTnI shares over 95% structural similarity with the human protein. Monitoring cTnI levels in canines is clinically important for diagnosing myocardial injury in veterinary medicine, particularly in aging dogs or those with underlying cardiac conditions. Moreover, validating the assay in both species supports its translational potential across human and veterinary health applications. Data were collected from both human and canine samples, and we applied machine learning algorithms to analyze these profiles. The objective was to determine whether the data could be used to accurately distinguish concentration ranges and assess assay consistency across diverse sample sets.

### 2.6 EDA Processing

We developed a machine learning pipeline to analyze temporal colorimetric signals from the assays, enabling classification of results into healthy and unhealthy ranges. This pipeline was independently applied to two distinct biological cohorts: (1) a canine dataset consisting of 18 samples (11 healthy, 7 unhealthy or elevated) and (2) a human dataset comprising 22 samples (13 healthy, 9 unhealthy) where healthy cTnI level is <40 ng L^−1^ and unhealthy is >40 ng L^−1^ for human [19, 61]; in canines, the corresponding threshold was 100 ng L^−1^ [62, 63], with values below indicating healthy status and above indicating cardiac injury. ELISA values were used as ground truth. Although the analytical methodology was consistent across both cohorts, all steps from feature engineering to model training and validation were executed independently. For canine cTnI, while standardized high‐sensitivity thresholds are less well‐established, multiple prior studies have reported upper reference limits near 100 ng L^−1^ in clinically normal dogs. Consistent with these reports, clinical data from the TAMU Veterinary School indicate that cTnI concentrations in healthy dogs consistently fall below 100 ng L^−1^. Accordingly, a conservative threshold of 100 ng L^−1^ was selected to differentiate normal and elevated canine samples for classification and comparative analysis. This strategy ensured species‐specific interpretability while preventing any risk of cross‐cohort data leakage. The pipeline was structured into three interdependent phases: preprocessing (See material and methods), exploratory data analysis (EDA), and modeling. Each phase was carefully designed to address the practical challenges posed by limited sample sizes and high‐dimensional feature spaces. The EDA phase functioned as a quality control mechanism and a crucial step for generating inductive hypotheses. Considering the limited sample size to the number of features (*n* ≪ *p*), it was imperative to implement dimensionality reduction before engaging in supervised modelling to mitigate the risks of overfitting and noise amplification.

**FIGURE 5:**
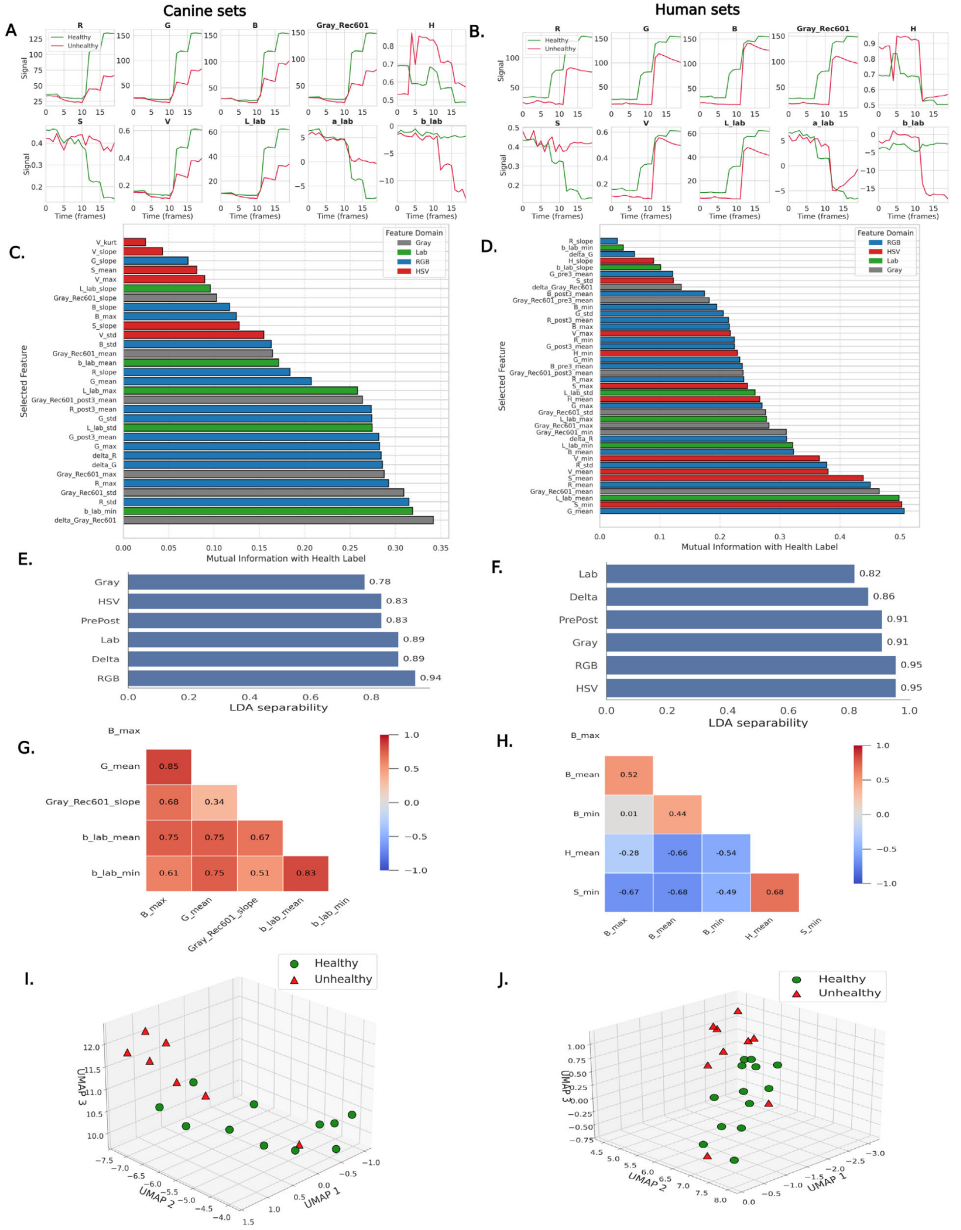
Comparative exploratory analysis of temporal color signals from the assay in canines (left) and humans (right). (A,B) Mean temporal trajectories for selected color channels in healthy (green) versus unhealthy (red) cohorts. Clear deviations are observed across RGB, Lab, and Gray_Rec601_ channels, indicating pathology‐associated dynamics. (C,D) Features retained after univariate selection (Mann–Whitney U, *p* < 0.05; Mutual Information *>*0.02), color‐coded by domain. (E,F) Domain‐level LDA separability scores reveal high discriminative capacity in RGB and delta features for canines, and HSV and grayscale for humans. (G,H) Correlation heatmaps of top features post‐pruning (*|r| ≥* 0.90), highlighting intra‐domain dependencies and multicollinearity. (I,J) Three‐dimensional UMAP embeddings of the final pruned feature set, demonstrating apparent clustering between healthy and unhealthy classes, supporting the hypothesis that color encodes clinically relevant information.

Before conducting formal univariate or multivariate analyses, we visualized the average temporal trajectories of representative color channels in healthy and unhealthy canines and humans (Figure 5A,B). These comparisons revealed distinct cohort‐specific dynamics, especially within the RGB and grayscale channels, wherein subjects classified as unhealthy demonstrated reduced signal variance. The channels extracted from HSV and Lab color spaces exhibited significant divergence, indicating a sensitivity to the underlying physiological states. The initial patterns observed served to inform and drive the subsequent hypothesis‐driven analyses.

#### 2.6.1 Univariate Screening

In order to assess the predictive value of specific features, we performed univariate analyses that contrasted healthy and unhealthy subjects across each cohort. The Mann–Whitney *U* test was employed to evaluate distributional differences, as it is a non‐parametric approach appropriate for biological data that frequently exhibit deviations from normality. The test statistic is articulated as follows:

(1) $$U=n_{1}n_{2}+\frac{n_{1}\left(n_{1}+1\right)}{2}-R_{1}$$

where *n*_1_ and *n*_2_ signify the sample sizes corresponding to the two distinct groups, *R*_1_ refers to the cumulative sum of ranks allocated to the observations within the first group. Features demonstrating statistically significant differences (*p* < 0.05) were preserved for further analysis. Nonetheless, the presence of statistical significance does not inherently ensure predictive relevance. In order to tackle this issue, we enhanced the hypothesis‐testing framework by incorporating mutual information (mi). This metric quantifies the extent of shared information between a specific feature *x* and the target label *y*. This approach effectively captures both linear and non‐linear dependencies. The computation of mi is as follows:

(2) $$mi\;\left(x,y\right)=\sum_{x,y}p\left(x,y\right)log\frac{p\left(x,y\right)}{p\left(x\right)p\left(y\right)}$$

where *p*(*x, y*) represents the joint probability distribution, while *p*(*x*) and *p*(*y*) denote the marginal distributions associated with the respective variables. The features were selected for downstream modelling solely based on their fulfilment of two critical criteria: statistical significance (*p* < 0.05) and information relevance (mi *>* 0.02). This dual‐filtering strategy guarantees identifying features that are statistically significant and possess substantive predictive value. The summary of the most significant retained features, organized by mutual information and distinguished by domain through color coding, is presented in Figure 5C,D for both species.

#### 2.6.2 Intragroup Multivariate Analysis

An initial categorization was conducted based on their respective origins: RGB, HSV, Lab, greyscale, delta, and temporal domains to examine the dynamics of features at the group level. The underlying reasoning was that the interdependencies among features within each group could potentially uncover synergistic patterns that are not apparent when examined at the univariate level. We performed a multivariate analysis of variance (MANOVA) for each group and species to evaluate whether the joint distribution of features exhibited significant variation across health classes. The results, which reached statistical significance, demonstrated a structured variation within these feature spaces. To evaluate the linear discriminative capabilities of each group, we utilized linear discriminant analysis (LDA) to obtain separability scores. The separability of feature domains derived from LDA is depicted in Figure 5E,F, showcasing distinct patterns: The analysis revealed that RGB and delta features exhibited the highest level of separability in canines. In contrast, HSV and RGB features were predominant in humans. Furthermore, Hotelling's *T* ^2^ statistic was employed to identify multivariate outliers, which may lead to bias in the classification boundaries.

We systematically evaluated all potential intra‐group feature pairs through logistic regression to discern interactions that enhance class discrimination. Feature pairs that produced an area under the receiver operating characteristic curve (AUC) of ≥0.85 were preserved. This methodology capitalizes on the potential for individually weak features to interact synergistically, thereby yielding robust predictors.

#### 2.6.3 Intergroup Refinement

Following the aggregation of high‐performing features identified across all groups, a Pearson correlation analysis was conducted to examine potential multicollinearity. Features exhibiting absolute correlation coefficients greater than |*r*| *>* 0.90 were systematically eliminated to maintain the model's interpretability and stability. Figure 5G,H depicts the remaining inter‐feature correlations as heatmaps, demonstrating the preservation of intra‐domain structure while effectively mitigating multicollinearity. A final MANOVA was conducted on the consolidated dataset to assess the discriminative capacity of the retained features. The analysis demonstrated statistically significant class separation (*p* < 0.01), thereby confirming the overall efficacy of the intergroup feature set. We utilized three‐dimensional embeddings generated through Uniform Manifold Approximation and Projection (UMAP) to assess class separability within the pruned feature space. This non‐linear manifold learning technique effectively maintains the integrity of local neighborhood structures while facilitating intuitive visualization. The canine and human cohorts analysis yielded embeddings that demonstrated clear clustering between healthy and unhealthy subjects. The UMAP projections derived from the refined feature space for both cohorts demonstrate clear visual class separability across three dimensions (Figure 5I,J).

### 2.7 Modelling

#### 2.7.1 Cross‐Validation Protocol

The modelling of each cohort was conducted independently through the application of leave‐one‐out cross‐validation (LOOCV). This rigorous resampling method is especially effective for datasets with low sample sizes (*n*). LOOCV optimizes training data by systematically excluding one sample for testing purposes while utilizing the remaining *n*−1 samples for training. This approach yields high‐fidelity and nearly unbiased estimates of generalization performance. The deterministic nature of this approach effectively mitigates the variance typically introduced by random splits in conventional *k*‐fold validation.

#### 2.7.2 Classifier Benchmarking

We comprehensively evaluated ten supervised learning algorithms that encompass a wide range of inductive biases and decision‐making mechanisms, including linear, probabilistic, kernel‐based, tree‐structured, ensemble, and neural architectures. The comprehensive list included Support Vector Classifier (SVC), Logistic Regression, k‐Nearest Neighbors (KNN), Gaussian Naive Bayes, Decision Tree, Random Forest, Extra Trees, Gradient Boosting, AdaBoost, and Multi‐Layer Perceptron (MLP). Each classifier underwent independent training and validation on canine and human datasets, employing the LOOCV protocol. The assessment of performance was conducted through the application of a clinically weighted utility score:

(3) $$\begin{array}{ccc} Utility\;Score & = & 0.4*Recall+\\ & & 0.2*F1\;Score+0.2*\;AUC_{ROC}+0.2*\;AUC_{PR} \end{array}$$

The weighting scheme prioritises recall to reduce false negatives, essential in clinical diagnostics due to the significant risks associated with overlooked pathological cases. The incorporation of both ROC and PR AUCs contribute to the robustness in the presence of potential class imbalance, while the F1 score offers a comprehensive harmonic balance between precision and recall.

#### 2.7.3 Hyperparameter Optimization

The hyperparameter search space was maintained uniformly across all optimization strategies (Grid Search, GA, PSO), with classifier‐specific parameters chosen under established domain conventions and the defaults provided by scikit‐learn. Table 1 below provides a comprehensive summary of the candidate hyperparameters and their respective value ranges. All models were initialized using random state = 42 before proceeding with hyperparameter optimization to ensure reproducibility.

The efficacy of a model is profoundly influenced by the arrangement of hyperparameters, which govern the learning dynamics, regularization, and decision thresholds. To systematically and independently optimize them, we utilized two global, gradient‐free search strategies: the genetic algorithm (GA) and particle swarm optimization (PSO). The GA operates as a population‐based evolutionary strategy, systematically selecting, recombining, and mutating candidate configurations following their fitness, which is quantified in this context by the utility score. The inherent stochastic characteristics facilitate investigating intricate, multimodal parameter spaces, rendering it appropriate for diverse classifiers. PSO is a metaheuristic approach inspired by physical principles, wherein candidate solutions are represented as particles navigating through the hyperparameter space. Each particle adjusts its velocity and position by weighing its best performance against the influence of neighboring particles, facilitating a dynamic adaptation process and convergence towards optimal regions. The implementation of PSO was conducted utilizing the opportunity (i.e. open source Python) framework, which supports flexible parameter bounds and facilitates integration with nested validation schemes.

The execution of each optimization method occurred within a nested cross‐validation framework. An inner 3‐fold stratified cross‐validation loop was meticulously integrated within the outer LOOCV cycle to mitigate the risks of information leakage and overfitting during the tuning process. The modal hyperparameter set, representing the configuration that achieved optimal performance most frequently across the LOOCV folds, was identified for each classifier and subsequently employed to retrain the final model utilizing the entire dataset. To evaluate the concrete effects of optimization, we conducted a comparative analysis of model performance before and after the tuning process. In the analysis of various classifier types, it was observed that hyperparameter optimization consistently enhanced the utility score in the case of humans. Notably, ensemble methods demonstrated the most significant absolute improvements, with a mean change in utility of approximately 0.06. It is important to note that default parameters frequently lead to the underutilization of model capacity or excessive regularization of the decision boundary, particularly in support vector classification (SVC) and multi‐layer perceptron (MLP). This observation underscores the need for systematic tuning in clinical settings with small sample sizes. Performance evaluation was carried out independently for both canine and human datasets. In both instances, tree‐based ensemble methods, specifically Random Forest, Extra Trees, and Gradient Boosting,s attained the highest utility scores post‐optimization, illustrating an exceptional equilibrium between sensitivity and specificity. The models yield interpretable feature importance rankings, thereby elucidating the predictive contributions of each color descriptor and temporal marker.

The classification performance for both human and canine datasets across all models and optimization strategies is comprehensively summarized in Tables S3 and S4. In the analysis of the human dataset, the Gradient Boosting and Decision Tree classifiers demonstrated the highest utility scores, reaching up to 89.8%. Notably, these classifiers maintained consistent sensitivity and precision throughout all iterations of the study. In the analysis of canine samples, the Gaussian Naïve Bayes and multi‐layer perceptron models exhibited superior performance, attaining utility scores exceeding 80%. The findings suggest that the engineered colorimetric and temporal characteristics exhibit a high degree of discrimination regarding health status, thereby affirming the practicality of employing real‐time color signal dynamics for precise and species‐translatable diagnostics. The evaluation of performance was carried out independently for both canine and human datasets. In both instances, tree‐based ensemble methods, specifically random forest, extra trees, and gradient boosting, attained the highest utility scores post‐optimization, illustrating an exceptional equilibrium between sensitivity and specificity. The models yield interpretable feature importance rankings, thereby elucidating the predictive contributions of each color descriptor and temporal marker.

The finalized models were serialized utilizing platform‐agnostic formats to ensure reproducibility and facilitate future integration into prototype diagnostic tools. The modular structure facilitates downstream deployment within clinical decision support systems or validation cohorts, requiring minimal modification.

**FIGURE 6:**
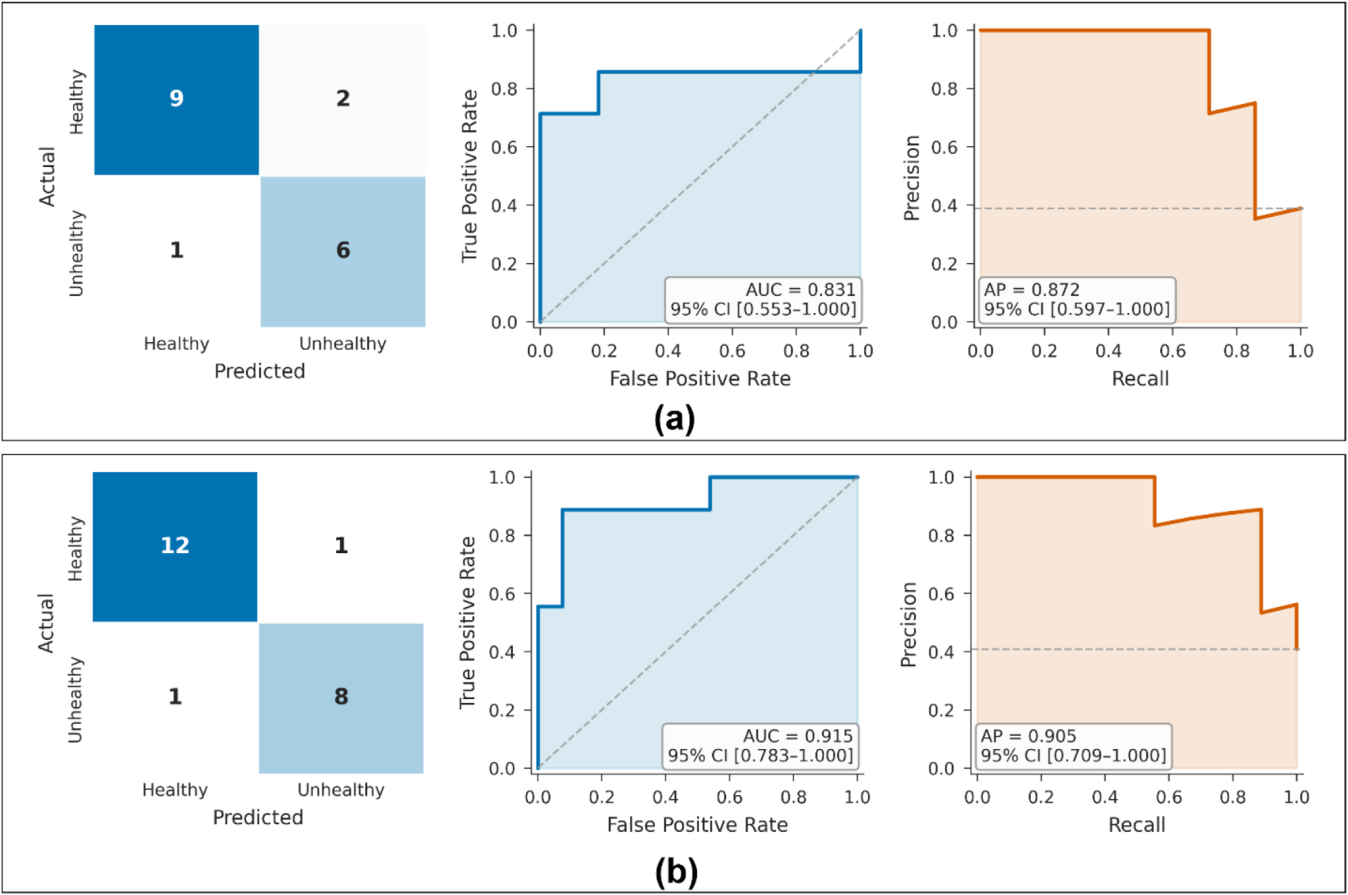
Classification performance of best models for canine and human datasets. (a) Gaussian Naive Bayes classifier for canine: confusion matrix (left), ROC curve (middle), and precision‐recall curve (right). The model demonstrates strong separability with ROC‐AUC = 0.831 (95% CI: 0.553–1.000) and PR‐AUC = 0.872 (95% CI: 0.597–1.000). (b) Gradient Boosting classifier for humans: confusion matrix (left), ROC curve (middle), and precision‐recall curve (right). The classifier shows high diagnostic performance with ROC‐AUC = 0.915 (95% CI: 0.783–1.000) and PR‐AUC = 0.905 (95% CI: 0.709–1.000).

To elucidate the diagnostic performance of the leading classifiers for each cohort, we provide confusion matrices in conjunction with ROC and precision–recall curves, as illustrated in Figure 6a,b. The analysis of the canine dataset revealed that the Gaussian Naïve Bayes classifier demonstrated notable separability, attaining an ROC‐AUC of 0.831 and a PR‐AUC of 0.872. In the analysis of the human dataset, the model that demonstrated superior performance was gradient boosting, attaining an ROC‐AUC of 0.915 and a PR‐AUC of 0.905. The findings substantiate the models' proficiency in precisely distinguishing between healthy and unhealthy individuals, while maintaining a low rate of false negatives, which is essential for the efficacy of clinical decision support systems.

In summary, this modelling pipeline, distinguished by comprehensive validation, clinically pertinent scoring, and biologically informed features demonstrates that the dynamics of color generated from assay embody diagnostic signals that are universally applicable across both canine and human populations. The consistent performance observed across various species underscores the potential of time‐lapse color analysis as a cost‐effective biomarker platform, facilitating advancements in health monitoring within translational contexts.

### 2.8 Blind Test Validation with Human Samples

To assess generalization performance, we conducted a prospective blind test using an independent cohort of 10 human serum samples (6 healthy, 4 unhealthy) withheld from model development. Ground truth labels from ELISA were concealed until after predictions were generated to eliminate potential bias from data leakage.

**FIGURE 7:**
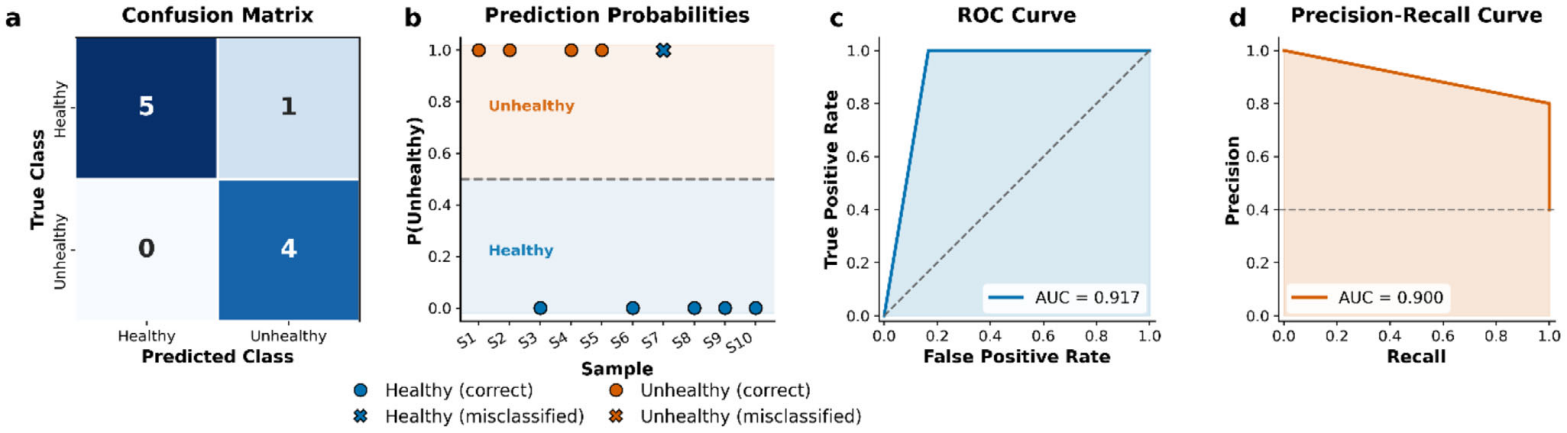
Blind test evaluation (*n* = 10). (a) Confusion matrix. (b) Prediction probabilities per sample; circles, correct predictions; crosses, misclassifications; blue, healthy; orange, unhealthy; dashed line, decision threshold. (c) ROC curve (AUC = 0.917). (d) Precision‐recall curve (AUC = 0.900); dashed line, baseline prevalence.

Blind test samples underwent identical preprocessing to the training cohort: color space transformation (RGB to HSV, CIE Lab, and Rec. 601 grayscale), followed by extraction of statistical features (mean, standard deviation, minimum, maximum, skewness, kurtosis, and linear slope) for each color channel, along with delta‐shift features capturing color transitions. The 32 features identified through multi‐stage selection during training (Table S5) were extracted in identical order, and the PSO‐optimized Gradient Boosting classifier (n_estimators = 130, learning_rate = 0.053, max_depth = 5) was applied without retraining. The classifier achieved 90% accuracy (9/10 correct; 4 true positives, 5 true negatives, 1 false positive, 0 false negatives). Given the limited sample size, we computed 95% confidence intervals using Wilson score intervals for proportions, bootstrap resampling (*n* = 2000) for complex metrics, and DeLong's method for AUC‐ROC (Table S6). The model demonstrated balanced performance with recall of 100.0% (95% CI: 51.0%–100.0%) and a specificity of 83.3% (95% CI: 43.6%–97.0%). ROC analysis yielded an AUC of 0.917 (95% CI: 0.753–1.000), and the precision‐recall curve achieved an AUC of 0.90 (95% CI: 0.70–1.00) (Figure 7c,d). The utility score, defined as the optimization objective during PSO hyperparameter tuning, was 92.1% (95% CI: 74.9%–100.0%). The wide confidence intervals reflect statistical uncertainty inherent to small‐sample validation; however, all lower bounds exceeded chance level, supporting the model's discriminative capacity pending validation with larger cohorts.

While these results demonstrate promising diagnostic performance, several limitations should be acknowledged. The relatively small sample sizes (*n* = 22 for model development; *n* = 10 for blind validation on humans) result in wide confidence intervals, as reflected in Tabless S3–S6. Although LOOCV maximizes training data usage and provides nearly unbiased estimates for small datasets, the statistical power remains limited. The wide CIs for the blind test (e.g., recall 95% CI: 51.0%–100.0%) reflect this uncertainty. Larger prospective validation cohorts are needed to narrow these intervals and confirm generalizability across diverse patient populations and clinical settings.

## 3 Conclusion

Building upon the same locked aptamer and HCR amplification framework developed in our previous SERRS‐based cTnI detection platform, we adapted the system into a simplified, reader‐free colorimetric assay by integrating catalytic DNAzymes for visual signal generation. This assay maintains the critical features of enzyme‐free amplification and target specificity while addressing practical limitations in speed, cost, and operational complexity. The importance of rapid and sensitive detection of cTnI cannot be overstated, especially for early diagnosis and monitoring of MI. In acute care, early rule‐in or rule‐out of MI is crucial to avoid unnecessary hospitalization, enable prompt initiation of therapy, and reduce patient burden. Current diagnostic workflows often rely on serial ECGs and cTnI testing using centralized laboratory equipment. However, ECG alone is only about 60% predictive and cannot definitively rule out MI, particularly in patients with atypical symptoms or comorbidities. High‐sensitivity cTnI testing is therefore essential for confidently stratifying risk in high‐risk or symptomatic individuals, especially within the first few hours of symptom onset. Furthermore, continuous or serial monitoring of cTnI levels provides critical insight into cardiac injury progression or resolution, particularly in post‐MI patients, those undergoing cardiac surgery, or individuals with chronic cardiovascular conditions. Unfortunately, ambulatory and pre‐hospital settings often lack access to high‐sensitivity troponin testing, due to reliance on bulky analyzers, need for trained personnel, and high per‐test costs. These logistical challenges limit timely decision‐making, particularly in rural areas, emergency transport, and field situations. To date, no commercially available test offers high‐sensitivity cTnI detection using a purely colorimetric format without external instrumentation. Our assay addresses this unmet need by offering a limit of detection of 0.25 ng/L, a wide dynamic range of 0.5–50 000 ng L^−1^, and a total turnaround time of just 25 min all with a simple color readout visible to the naked eye. This makes the platform especially valuable for decentralized and low‐resource environments where rapid, cost‐effective diagnostics are urgently needed. Importantly, the estimated total material cost of our colorimetric assay is approximately $2 per test (Table S7), substantially lower than commercial high‐sensitivity cTnI platforms such as the Abbott i‐STAT (∼$20–25 per cartridge) or the LSI PathFast system (∼$15–20 per test). Beyond its cost‐effectiveness, the assay demonstrates high stability and reproducibility across multiple assays and sample types, including human and canine serum, with minimal inter‐assay variability. The DNAzyme‐based amplification and modular aptamer design maintain consistent performance under ambient conditions, underscoring its robustness for decentralized, point‐of‐care applications. Coupled with rapid turnaround, equipment‐free readout, and minimal operational complexity, these features highlight the practical advantages of our platform over conventional commercial systems, making it well‐suited for low‐resource, ambulatory, pre‐hospital, and veterinary diagnostic settings. In addition to human diagnostics, the assay has potential utility in veterinary applications, such as in veterinary teaching hospitals and animal clinics. Given the >99% sequence and structural similarity between canine and human cTnI, our assay successfully detected cTnI in canine serum samples, offering a translational bridge for veterinary cardiac biomarker testing a currently underserved area in point‐of‐care diagnostics. In conclusion, our colorimetric DNAzyme‐based assay represents a powerful advancement in accessible cardiac diagnostics, offering ultrasensitive detection, broad dynamic range, rapid turnaround, and equipment‐free readout. Its modular design also allows for adaptability to other biomarkers simply by altering the aptamer sequence. With continued development, this platform holds strong potential to enable affordable, real‐time cardiac health monitoring in emergency, ambulatory, rural, and veterinary settings paving the way for truly decentralized, next‐generation cardiovascular diagnostics.

## 4 Material and Methods

### 4.1 Materials

Cardiac troponin I (cTnI) used in the experiments was obtained from GenScript (Piscataway, NJ, USA), and all DNA oligonucleotides were synthesized and purified by Integrated DNA Technologies (IDT, Coralville, IA, USA). Chemicals, including streptavidin and all general reagents, were purchased from Millipore Sigma. Magnetic particles (Dynabeads) were obtained from Invitrogen. cTnI‐free human serum was acquired from HyTest Ltd. (Turku, Finland). A commercial ELISA kit for cTnI quantification was obtained from Abcam (Cambridge, UK). Canine serum samples were collected under TAMU IACUC protocol  (IACUC 2020‐0193 CA, IACUC 2024‐0302 CA). Human patient serum samples were provided by the Department of Bioengineering at the University of California, Los Angeles (UCLA), under institutional review board (IRB) approval (Protocol #20‐002084‐AM‐00001).

### 4.2 TapeStation Analysis

To evaluate the amplification efficiency and product distribution of linear and hyperbranched HCR, Agilent TapeStation analysis was performed using the High Sensitivity D1000 ScreenTape system. The aptamer (1 µM) was incubated with 50 ng L^−1^ of cTnI. Then, 1 µM each of H1 and H2 hairpins was added, followed by a 30‐minute incubation to allow for linear hybridization chain reaction. For the hyperbranched HCR, 1 µM of locked aptamer was incubated with 50 ng L^−1^ cTnI along with four hairpins. Control samples were prepared using nuclease‐free water instead of cTnI. Reaction products from both linear HCR (involving H1 and H2) and hyperbranched HCR (involving all four hairpins) were collected and diluted appropriately in nuclease‐free water. A 2 µL aliquot of each sample was mixed with 2 µL of D1000 sample buffer, vortexed, and briefly centrifuged. Electrophoretic analysis was carried out according to the manufacturer's protocol. Fragment size distribution and smear profiles were used to compare the relative lengths and complexity of HCR amplicons generated under linear and branched conditions.

### 4.3 AFM Imaging

The mica which served as the substrate of the DNA was incubated in toluene (Sigma Aldrich, St. Louis, Missouri, USA, Cat. No.: 244511) with 0.1 µM (3‐Aminopropyl) triethoxysilane (Sigma Aldrich, Missouri, St. Louis, USA, Cat. No.: 440140) in room temperature for 4 h. Then the −NH_2_ functionalized mica was further incubated in methyl iodide for 1 h, washed with ddH_2_O twice and dried. 0.01 ng mL^−1^ of DNA product was purified and desalted with Nanosep centrifugal filter (Omega modified PES, MWCO: 3K, Cytiva, Marlborough, Massachusetts, USA, Cat. No.: OD003C33). Purified DNA products were eluted in a buffer containing 20 mM Tris−HCl, 20 mM NaCl, and 5 mM EDTA and then incubated on the surface of the modified mica for 1 h, followed by ddH_2_O washing. The AFM images of HCR products on the modified mica were captured by Bruker Dimension Icon AFM (Bruker, Billerica, Massachusetts, USA) in tapping mode (Tapping frequency: 150 Hz). AFM imaging was conducted in air‐tapping mode across the mica surface, with drive frequencies adjusted between 5 and 500 kHz. After image flattening, DNA contour lengths were measured using ImageJ following a protocol adapted from Lysetska et al.

### 4.4 Hemin DNAzyme Preparation

To incorporate hemin into the DNAzyme‐containing hairpin structures for colorimetric detection, the hairpin‐DNAzyme oligonucleotides were first snap‐cooled to ensure proper folding. Briefly, the hairpin DNAzyme strands were heated to 95°C for 5 min and immediately placed on ice for 10 min to facilitate correct secondary structure formation. Following this, the folded hairpin‐DNAzyme constructs (1 µM final concentration for each) were prepared in a total reaction volume of 30 µL using a colorimetry buffer composed of 25 mM Tris‐HCl (pH 7.6), 0.5 M NaCl, 20 mM KCl, 0.03% Triton X‐100, and 1% DMSO. The potassium concentration was optimized to support G‐quadruplex formation, which is essential for the catalytic activity of the DNAzyme‐hemin complex. Hemin was added to the mixture at a final concentration of 1 µM. The reaction was then incubated at room temperature for 2 h to allow efficient incorporation of hemin into the folded DNAzyme structures, enabling peroxidase‐like catalytic activity for downstream colorimetric detection. To elucidate the role of monovalent cations in G‐quadruplex folding and DNAzyme activity, three ion‐defined colorimetric buffers were prepared: (i) a Na^+^‐only buffer containing NaCl without KCl, (ii) a K^+^‐only buffer containing KCl without NaCl, and (iii) a mixed Na^+^/K^+^ buffer containing both salts. All buffers were prepared using 25 mM Tris–HCl (pH 7.6), supplemented with 0.03% Triton X‐100 and 1% DMSO. These conditions enabled direct comparison of ion‐dependent DNAzyme folding and catalytic performance while maintaining identical pH and solvent composition across experiments.

### 4.5 Determination of the Assay Sensitivity

Streptavidin‐coated magnetic particles (MPs, 100 µg) were washed three times with washing buffer containing 25 mM Tris, 150 mM NaCl, pH 7.2, 0.1% BSA, and 0.05% Tween‐20. Biotinylated aptamers were snap‐cooled by heating at 95 °C for 7 min followed by immediate cooling on ice, and then dissolved in 1× binding buffer (10 mM Tris, 1 mM EDTA, 1000 mM NaCl). The aptamer solution was incubated with the washed MPs overnight at 4°C with gentle mixing to enable conjugation via biotin‐streptavidin interaction. After incubation, the aptamer‐MP complexes were washed with 1× binding buffer and subsequently blocked using 1% BSA‐biotin solution for 45 min at room temperature to prevent nonspecific binding. The complexes were then washed and resuspended in high‐salt binding buffer supplemented with sodium phosphate. cTnI was prepared at the desired concentrations in binding buffer (20 mM Tris, pH 7.6, 500 mM NaCl, 5 mM KCl, 2 mM MgCl_2_, 1 mM CaCl_2_) and added to the aptamer‐MP complexes. Following that, Hemin‐preincubated four hairpins (final concentration 1 µM each) were then introduced to initiate a hybridization chain reaction (HCR), and the reaction mixture was incubated for 20 min. The resultant complexes were washed thoroughly with sodium phosphate and sodium chloride‐containing buffer and resuspended in colorimetric buffer. A 10 mM hemin stock solution was prepared in DMSO and stored at −20°C in the dark. Hemin incorporation into hairpins was performed by preparing hemin in a colorimetric buffer, adding it to the DNAzyme‐containing hairpins, and incubating the mixture for 2 h at room temperature. ABTS and H_2_O_2_ were then added, and color development was observed within 5 min. For serum‐spiked samples, human serum was heat‐inactivated at 56°C for 10 min to reduce immunogenicity and aliquoted for storage. Desired concentrations of cTnI were spiked into the heat‐inactivated serum. Preincubated hairpins (final concentration 2 µM each) were added and incubated for 20 min, and subsequent assay steps were performed as described above. The magnetic particle–HCR–DNAzyme complexes were then resuspended in colorimetric buffer. For colorimetric detection, freshly prepared ABTS (2 mM) and H_2_O_2_ (5 µM) in 0.15 M citrate‐phosphate buffer (pH 5.5) were added. The intensity of the developed green color, produced by DNAzyme‐mediated oxidation of ABTS, was used to assess the presence and relative concentration of cTnI in each clinical sample.

### 4.6 Assay with Clinical Sample

To evaluate the clinical applicability of the developed colorimetric assay, a total of 22 human patient serum samples and 18 canine serum samples were analyzed. Human serum samples were obtained from the University of California, Los Angeles (UCLA), and canine samples were provided by the Texas A&M University (TAMU) College of Veterinary Medicine. The serum was aliquoted into small volumes and stored at −80°C until use. Prior to analysis, samples were thawed on ice and used directly without further dilution unless otherwise specified. Each sample was subjected to the same assay protocol as described for buffer‐based experiments. Specifically, hemin incubated four hairpins (final concentration 1 µM each), previously annealed with hemin to form a catalytically active DNAzyme complex, were added to the serum samples obtained from patients or canine samples. The reaction mixtures were incubated for 20 min at room temperature to allow target recognition by the aptamer and subsequent hybridization chain reaction (HCR)‐based signal amplification. Following incubation, the resulting complexes were washed thoroughly with buffer containing sodium phosphate and sodium chloride to remove unbound components. The magnetic particle–HCR–DNAzyme complexes were then resuspended in colorimetric buffer. For colorimetric detection, freshly prepared ABTS (2 mM) and H_2_O_2_ (5 µM) in 0.15 M citrate‐phosphate buffer (pH 5.5) were added. The intensity of the developed green color, produced by DNAzyme‐mediated oxidation of ABTS, was used to assess the presence and relative concentration of cTnI in each clinical sample. This assay protocol was consistently applied to both human and canine samples, and the data obtained were used to validate the performance of the biosensor across species and biological matrices. Colorimetric responses were visually assessed and digitally recorded for subsequent comparative and quantitative analysis.

### 4.7 Machine Learning

We developed a comprehensive machine learning pipeline to assess the potential of temporal color signals from the assay in the presence of cTnI. We implemented it independently across two distinct biological cohorts: (1) a canine dataset consisting of 18 samples (11 healthy and 7 unhealthy), and (2) a human dataset comprising 22 samples (13 healthy and 9 unhealthy). The analytical process was methodologically consistent across both cohorts; however, all computations, ranging from feature engineering to model training and validation were executed independently. This approach was essential to maintain species‐specific interpretability and to prevent any potential cross‐cohort contamination.

The pipeline was meticulously organized into three interrelated phases: Preprocessing, EDA, and Modelling. The progression through each stage was driven by practical limitations, such as the constraints imposed by small sample sizes, and informed by established domain expertise. The design sought to enhance model performance while guaranteeing that statistical inferences were robust, biologically relevant, and applicable across various cohorts.

### 4.8 Preprocessing

Colorimetric signals were obtained as timestamped RGB sequences via a calibrated optical device placed after the assay was done from the clinical sample. The raw RGB values demonstrate a significant sensitivity to lighting conditions and sensor response fluctuations, failing to align with perceptual or physiological color axes. As a result, we systematically mapped each signal into three distinct complementary color spaces: HSV, which allows for the capture of perceptual hue and saturation variations; CIELAB, chosen for its perceptual uniformity and effective luminance separation; and Grayscale (Rec. 601), employing the following formula:

(4) $$Gray_{Rec601}=0.299R+0.587G+0.114B$$

emphasizing lightness contrast without chromatic influence.

Statistical summaries were meticulously extracted from each color channel, encompassing mean, standard deviation, skewness, and kurtosis. Furthermore, temporal regression slopes were employed to evaluate linear trends, adeptly reflecting both stable color levels and gradual changes over time. Nonetheless, pathology often induces sudden changes, including inflammatory flares or vascular events, which require the deployment of a delta shift detector:

(5) $$\Delta_{x}=\frac{1}{k}\sum_{t=t_{0}+1}^{t_{0}+k}x_{t}-\;\frac{1}{k}\sum_{t=t_{0}\;-k}^{t_{0}-1}x_{t}$$

In this context, *t*_0_ is identified as the frame exhibiting the highest temporal contrast in grayscale, while *k* = 3 specifies the parameters for the smoothing window. This operator assesses the direction and magnitude of transient events posited to hold biological significance.

As a culmination of the aforementioned transformations, the final feature vector for each subject consisted of 84 dimensions, meticulously extracted from the transformed time‐series signals. The identified features were systematically categorized into three principal groups. The first group consisted of color statistics encompassing 70 descriptors that capture the mean, variance, extrema, skewness, kurtosis, and temporal slopes across ten perceptual channels (*R, G, B, GrayRec*601*, H, S, V, L_lab_, a_lab_, b_lab_*). The second group of delta shift features consisted of 12 measures that reflect abrupt transient events based on localized pre/post averages surrounding the most significant grayscale transition, and the third group of metadata included a binary health label and an optional subject identifier. Implementing this structured feature representation facilitated a uniform approach to statistical evaluation and classification, independent of variations in raw sequence length or temporal complexity.

The result was a fixed‐length feature vector per subject (**x** *_i_* ∈ *R^p^*), forming the matrices **X** *_dog_* ∈ *R* ^18^ *^×p^* and **X** *_human_* ∈ *R* ^22^ *^×p^*, each paired with binary labels *y* ∈ {0, 1} indicating health status. This transformation laid the groundwork for unbiased comparison, regardless of original time‐series length or species.

### 4.9 Statistical Analysis and Sensitivity Testing

Sensitivity and specificity of the assay were evaluated using both buffer‐ and human serum–spiked samples. For each sample type, five different concentrations (including the negative control) were tested, with three repeated measurements per concentration, resulting in a total of 15 reactions. The limit of detection for cTnI was calculated as the concentration corresponding to a signal intensity equal to the mean negative control intensity plus three times the standard deviation of the blank signal (calculated across the three repeats for each concentration). All measurements were performed in triplicate (N = 3) for each concentration. Data are presented as mean ± standard deviation (SD). Statistical significance was assessed using one way ANOVA LSD method, and all analyses were performed using SPSS software. *p*‐values <0.05 (*) were considered statistically significant.

## Conflicts of Interest

The authors (ST, GC, SM) have a pending patent application on this technology.

## Supporting information




**Supporting File**: smll72762‐sup‐0001‐SuppMat.docx.

## Data Availability

The data that support the findings of this study are available from the corresponding author upon reasonable request.
