# Supplementary material for: High Sensitivity Cardiac Troponin I Detection via MP‐Locked Aptamer and Multimeric DNAzyme‐Coupled Hyperbranched Hybridization Chain Reaction
Source: Small. 2026 Feb 23;22(20):e12096. doi: 10.1002/smll.202512096 (PMC13054452; doi:10.1002/smll.202512096)
Supplement: Supplementary file 1 — Supporting File: smll72762‐sup‐0001‐SuppMat.docx. [file SMLL-22-e12096-s001.docx]

**High Sensitivity Cardiac Troponin I Detection via MNP-Locked Aptamer and Multimeric DNAzyme-Coupled Hyperbranched Hybridization Chain Reaction**

# Sayantan Tripathy^a,b^, Sahil Sharma^c^, Ng Ka Wai^a^, Siddhant Jaitpal ^a^, Anthony Dongchau ^a^, Saugat Bhattacharyya^c^, Sonya R Wesselowski^d^, Ashley B. Saunders^d^, Gerard L. Coté^a,b,e^*, Samuel B. Mabbott^a,b^*

^a^ Department of Biomedical Engineering, Texas A&M University, 600 Discovery Drive, College Station, TX, 77840-3006, USA

^b^ Center for Remote Health Technologies and Systems, Texas A&M University, 600 Discovery Drive, College Station, TX, 77840-3006, USA

^c^  School of Computing, Engineering and Intelligent Systems, Ulster University, Londonderry - BT48 7JL, Northern Ireland, United Kingdom

^d^ Department of Small Animal Clinical Sciences, College of Veterinary Medicine & Biomedical Sciences, Texas A&M University, College Station, TX 77843-4474, USA

^e^ Department of Electrical and Computer Engineering, Texas A&M University, College Station, TX, 77840, USA

*Corresponding author: [smabbott@tamu.edu](mailto:smabbott@tamu.edu)

| **Figures** | Page no |
| --- | --- |
| Figure S1: Thermodynamic Profile of Locked Aptamer | 2 |
| Figure S2: Folding of locked aptamer and its thermodynamic stability | 3 |
| Figure S3: Effect of potassium ions on colorimetry | 4 |
| Figure S4: AFM morphology analysis. | 5 |
| Figure S5: Colorimetric optimization of DNAzyme sequence | 6 |
| Figure S6: Colorimetric Assay Sensitivity Determination of cTnI in aqueous solution using Linear HCR (1^st^ generation) | 7 |
| Figure S7: Colorimetric Assay Sensitivity Determination of cTnI in serum spiked sample solution using Linear HCR (1^st^ generation) | 8 |
| Figure S8: The hyperbranched HCR system was evaluated for its ability to enhance the sensitivity of cTnI detection in serum-spiked samples | 9 |
| Figure S9: Updated Protocol for cTnI Quantification Using a Colorimetric Reade | 10 |
| Figure S10: Color Matrix Formation and ΔG Calculation for Colorimetric Signal Quantification | 11 |
| Figure S11: Specificity test with our assay. | 11 |
| **Tables** | |
| Table S1: Sequences of oligonucleotides | 12 |
| Table S2: Hyperparameter search space for model tuning (used in GridSearch, GA, and PSO) | 13 |
| Table S3: Classification results for the canine dataset (n=18) using 10 machine learning models with 95% CI | 14 |
| Table S4: Classification results for the human dataset (n=22) using 10 machine learning models with 95% CI | 16 |
| Table S5: Selected features (n=32) for classification model | 18 |
| Table S6: Blind test classification performance with 95% confidence intervals | 19 |
| Table S7: Cost estimation of our assay | 19 |

**Figure S1** Folding of locked aptamer and its thermodynamic stability A, Schematic representation of probable structure of locked aptamer sequence. B, Folding thermodynamics

**Figure S2**: Probable complex formation among the hairpins

**Figure S3:** AFM morphology analysis. morphological evidence of the progressive structural complexity of HCR products shows in hyperbranched HCR (Hyperbranched (2^nd^ generation) HCR) the population of free product is decreasing which indicates branched structure.

**Figure S4: A,**Effect of potassium ions on colorimetry. Optimization studies revealed that **20 mM KCl** provides the most effective enhancement of peroxidase-like activity, yielding maximum color development. Higher concentrations showed no further improvement and, in some cases, led to background signal elevation, making 20 mM KCl the optimal concentration for DNAzyme-mediated colorimetric detection. B, Role of sodium ions in colorimetry. C, Investigation of catalytic activity of hairpins. D, Stabilization of hairpin in 12% Native PAGE in presence of hemin and citrate phosphate buffer.

**Figure S5:** Colorimetric optimization of DNAzyme sequence. A mixture of G-quadruplex and trimeric triplex DNA structures produced the most intense colorimetric signal, with visible color development occurring within 5 minutes, indicating synergistic enhancement of DNAzyme activity.

**Figure S6**: Colorimetric Assay Sensitivity Determination of cTnI in aqueous solution using Linear HCR (1^st^ generation): To evaluate the sensitivity of the linear HCR-based colorimetric assay, varying concentrations of the target analyte (e.g., cTnI) were tested using the first-generation HCR system. The signal amplification was visualized by a DNAzyme-mediated colorimetric reaction, and quantitative assessment of color intensity was performed via RGB image analysis. The assay demonstrated a clear, concentration-dependent increase in color intensity, with reliable detection down to 0.05 ng/L, indicating the limit of detection (LOD). This high sensitivity is attributed to the efficient signal amplification enabled by the linear HCR mechanism, which produces extended nicked double-stranded DNA polymers that enhance DNAzyme loading and catalytic activity.

**Figure S7**: Colorimetric Assay Sensitivity Determination of cTnI in serum spiked sample solution using Linear HCR (1^st^ generation): A, Colorimetric signal and B, corresponding RGB analysis reveal that the current linear HCR system enables detection of cTnI down to 500 ng/L in serum-spiked samples. However, this detection limit is significantly higher than the clinical cutoff of 50 ng/L, indicating that additional signal amplification strategies such as hyperbranched HC R are necessary to achieve clinically relevant sensitivity.

Figure S8:

**Figure S8**: The hyperbranched HCR system was evaluated for its ability to enhance the sensitivity of cTnI detection in serum-spiked samples. RGB analysis of the resulting colorimetric signals demonstrated a significantly improved limit of detection, with reliable detection down to 0.5 ng/L well below the clinical cutoff of 50 ng/L. However, at higher analyte concentrations, the assay exhibited reduced resolution and poor quantitative discrimination due to the excessive signal amplification inherent to the hyperbranched architecture. This saturation effect limits the ability to distinguish between medium and high cTnI levels, suggesting that while hyperbranched HCR is well-suited for highly sensitive detection, it may require tuning or normalization strategies for accurate quantification across a broader dynamic range.

**Figure S9**: Updated Protocol for cTnI Quantification Using a Colorimetric Reader. An optimized readout protocol was developed to improve quantification of cTnI in serum-spiked samples using magnetic bead-based separation. Specifically, performing magnetic separation 4 minutes after initiating the colorimetric reaction was found to enhance signal clarity and reproducibility. This timing adjustment allows for more consistent DNAzyme-substrate interaction and minimizes background interference, enabling accurate discrimination across a broad dynamic range of cTnI concentrations.

**Figure S10:** Color Matrix Formation and ΔG Calculation for Colorimetric Signal Quantification:

Gray = 0.2989 × R + 0.5870 × G + 0.1140 × B.

The resulting grayscale intensity values were then used to calculate ΔG (change in gray value), defined as the difference between the signal (sample) and background (blank or negative control) grayscale intensities. This ΔG value serves as a quantitative metric for assessing color change and enables consistent comparison of assay performance across different experimental conditions. This method will be uniformly applied across all colorimetric assays in the study to ensure analytical consistency and reproducibility.

**Table S1:** Oligonucleotide Sequences (hairpin 3 and hairpin 4 are from^[1]^ [**10.1039/C8AN02284D**](https://doi.org/10.1039/C8AN02284D))

| Name of the sequences | Sequences (5’-3’) |
| --- | --- |
| Aptamer | CGTGCAGTACGCAACCTTTCTCATGCGCTGCCCCTCTT |
| Modified locked Aptamer | TTTTTTTTTTTTCGTGCAGTACGCAACCTTTCTCATGCGCTGCCCCTCTTAAAAAAAAGCAGATCCTAAGCCGCACCCAATTAAGGGGCAGCGCATGAGAAAGGTTCCGTA |
| Hairpin 1 | AATTGGGTGCGGCTTAGGATCTGCACAAAGGCAGATCCTAAGCCGCACTGGGAAGGGAGGGTGGGAAGGGAGGGTGGGAAGGGAGGG |
| Hairpin 2 | GCAGATCCTAAGCCGCACCCAATTGTGCGGCTTAGGATCTGCCTTTGT AGAAGAAGGTGTTTAAGTA |
| Hairpin 3 | AGGGCGGGTGGGTGTTTAAGTTGGAGAATTGTACTTAAACACCTTCTTCTTGGGT |
| Hairpin 4 | TGGGTCAATTCTCCAACTTAAACTAGAAGAAGGTGTTTAAG |

**Table S2**: Hyperparameter search space for model tuning (used in GridSearch, GA, and PSO)

**Classifier Hyperparameters and Values**

SVC *C* ∈ {0*.*1*,* 1*,* 10*,* 100}; kernel ∈ {*linear, rbf* }; gamma ∈ {*scale, auto*}

Logistic Regression *C* ∈ {0*.*1*,* 1*,* 10}; penalty ∈ {*l*1*, l*2}

KNN *k* ∈ {1*,* 3*,* 5}; weights ∈ {*uniform, distance*}

Decision Tree max depth ∈ {3*,* 5*,* 10}; min samples split ∈ {2*,* 4}

Random Forest *n estimators* ∈ {20*,* 50*,* 100}; max depth ∈ {2*,* 3*,* 5*,* 10}; min split ∈ {2*,* 3*,* 4}

Extra Trees *n estimators* = 100; max depth ∈ {5*,* 10}; min split ∈ {2*,* 4}

Gradient Boosting *n estimators* ∈ {100*,* 200}; learning rate ∈ {0*.*05*,* 0*.*1}; max depth ∈ {3*,* 5}

AdaBoost *n estimators* ∈ {50*,* 100}; learning rate ∈ {0*.*1*,* 1*.*0}

MLP hidden layers ∈ {(5)*,* (10)*,* (20)*,* (50)*,* (100)}; alpha ∈ {0*.*0001*,* 0*.*0005*,* 0*.*001}

Gaussian NB None (no hyperparameters)

**Table S3:** Classification results for the canine dataset (n=18) using 10 machine learning models optimized via Grid Search, Genetic Algorithm (GA), and Particle Swarm Optimization (PSO). All metrics are presented as percentages with 95% confidence intervals in brackets. The utility score (0.4×recall + 0.2×F1 + 0.2×AUC-ROC + 0.2×AUC-PR) prioritizes recall for clinical screening. Gaussian Naïve Bayes achieved the highest utility score (84.4%) consistently across all optimization strategies.

| **Grid Search** | | | | | | | | |
| --- | --- | --- | --- | --- | --- | --- | --- | --- |
| **Model** | **Accuracy** | **Precision** | **Recall** | **F1 Score** | **AUC-ROC** | **AUC-PR** | **Utility** | **Best Parameters** |
| SVC | 77.8 [54.8-91.0] | 80.0 [33.3-100.0] | 57.1 [25.0-84.2] | 66.7 [25.0-90.9] | 72.7 [42.8-100.0] | 76.8 [44.4-100.0] | 66.3 [31.3-94.3] | C=1, γ=scale, kernel=rbf |
| RandomForest | 66.7 [43.7-83.7] | 57.1 [16.7-100.0] | 57.1 [25.0-84.2] | 57.1 [18.2-82.4] | 70.1 [37.6-100.0] | 76.4 [42.9-100.0] | 63.8 [28.1-92.1] | depth=2, split=2, n=50 |
| LogisticRegression | 61.1 [38.6-79.7] | 50.0 [16.7-83.3] | 71.4 [35.9-91.8] | 58.8 [22.2-82.4] | 68.8 [39.3-98.4] | 53.4 [30.6-96.4] | 66.1 [33.1-91.8] | C=0.1, penalty=l1 |
| KNeighbors | 77.8 [54.8-91.0] | 80.0 [33.3-100.0] | 57.1 [25.0-84.2] | 66.7 [24.9-90.9] | 81.8 [56.3-100.0] | 83.5 [51.3-100.0] | 69.0 [35.6-96.0] | k=5, weights=uniform |
| DecisionTree | 72.2 [49.1-87.5] | 66.7 [20.0-100.0] | 57.1 [25.0-84.2] | 61.5 [20.0-87.5] | 71.4 [46.8-96.1] | 69.8 [25.0-88.9] | 60.9 [27.2-86.8] | depth=3, split=2 |
| GradientBoosting | 66.7 [43.7-83.7] | 57.1 [16.7-100.0] | 57.1 [25.0-84.2] | 57.1 [18.2-82.4] | 63.6 [34.2-93.1] | 54.6 [27.2-90.3] | 59.6 [25.3-86.6] | lr=0.05, depth=3, n=100 |
| AdaBoost | 77.8 [54.8-91.0] | 80.0 [33.3-100.0] | 57.1 [25.0-84.2] | 66.7 [25.0-92.3] | 66.2 [34.4-98.1] | 62.9 [29.2-95.7] | 62.7 [26.7-91.0] | lr=0.1, n=50 |
| **GaussianNB** | **83.3 [60.8-94.2]** | **75.0 [40.0-100.0]** | **85.7 [48.7-97.4]** | **80.0 [50.0-100.0]** | **83.1 [55.3-100.0]** | **87.2 [59.7-100.0]** | **84.4 [53.4-100.0]** | **Default** |
| ExtraTrees | 72.2 [49.1-87.5] | 66.7 [20.0-100.0] | 57.1 [25.0-84.2] | 61.5 [22.2-88.9] | 70.1 [36.2-100.0] | 77.4 [42.9-100.0] | 64.8 [28.3-93.3] | depth=5, split=4, n=100 |
| MLP | 88.9 [67.2-96.9] | 100.0 [100.0-100.0] | 71.4 [35.9-91.8] | 83.3 [46.1-100.0] | 84.4 [56.6-100.0] | 88.8 [62.5-100.0] | 80.0 [46.7-100.0] | α=0.0001, layers=(50,) |
| **Genetic Algorithm (GA)** | | | | | | | | |
| **Model** | **Accuracy** | **Precision** | **Recall** | **F1 Score** | **AUC-ROC** | **AUC-PR** | **Utility** | **Best Parameters** |
| SVC | 55.6 [33.7-75.4] | 33.3 [0.0-100.0] | 14.3 [2.6-51.3] | 20.0 [0.0-53.3] | 61.0 [29.7-92.4] | 65.6 [31.5-92.4] | 35.3 [12.7-61.4] | C=100, kernel=rbf, γ=scale |
| RandomForest | 72.2 [49.1-87.5] | 66.7 [20.0-100.0] | 57.1 [25.0-84.2] | 61.5 [22.2-88.9] | 69.5 [38.0-100.0] | 72.6 [40.6-96.7] | 63.8 [28.3-91.3] | n=100, depth=5, split=4 |
| LogisticRegression | 66.7 [43.7-83.7] | 55.6 [20.0-87.5] | 71.4 [35.9-91.8] | 62.5 [25.0-85.7] | 71.4 [37.2-100.0] | 79.2 [44.4-100.0] | 71.3 [33.9-95.7] | C=10, penalty=l2 |
| KNeighbors | 77.8 [54.8-91.0] | 71.4 [33.3-100.0] | 71.4 [35.9-91.8] | 71.4 [30.8-94.1] | 81.8 [59.7-100.0] | 79.0 [36.5-100.0] | 73.8 [40.1-97.5] | k=1, weights=uniform |
| DecisionTree | 72.2 [49.1-87.5] | 66.7 [20.0-100.0] | 57.1 [25.0-84.2] | 61.5 [20.0-87.5] | 71.4 [46.8-96.1] | 69.8 [25.0-88.9] | 60.9 [27.2-86.8] | depth=10, split=4 |
| GradientBoosting | 66.7 [43.7-83.7] | 57.1 [16.7-100.0] | 57.1 [25.0-84.2] | 57.1 [18.2-82.4] | 68.8 [42.7-95.0] | 49.3 [28.3-92.5] | 59.5 [27.7-86.7] | n=100, lr=0.05, depth=3 |
| AdaBoost | 77.8 [54.8-91.0] | 80.0 [33.3-100.0] | 57.1 [25.0-84.2] | 66.7 [25.0-92.3] | 79.2 [55.4-100.0] | 79.6 [48.4-100.0] | 68.1 [34.9-96.0] | n=100, lr=0.1 |
| GaussianNB | 83.3 [60.8-94.2] | 75.0 [40.0-100.0] | 85.7 [48.7-97.4] | 80.0 [50.0-100.0] | 83.1 [55.3-100.0] | 87.2 [59.7-100.0] | 84.4 [53.4-100.0] | Default |
| ExtraTrees | 72.2 [49.1-87.5] | 66.7 [20.0-100.0] | 57.1 [25.0-84.2] | 61.5 [22.2-88.9] | 70.1 [36.2-100.0] | 77.4 [42.9-100.0] | 64.8 [28.3-93.3] | n=100, depth=5, split=4 |
| MLP | 72.2 [49.1-87.5] | 62.5 [25.0-100.0] | 71.4 [35.9-91.8] | 66.7 [28.6-90.0] | 72.7 [45.5-99.9] | 70.6 [34.5-96.7] | 70.9 [35.7-95.0] | layers=(50,), α=0.0001 |
| **Particle Swarm Optimization (PSO)** | | | | | | | | |
| **Model** | **Accuracy** | **Precision** | **Recall** | **F1 Score** | **AUC-ROC** | **AUC-PR** | **Utility** | **Best Parameters** |
| SVC | 72.2 [49.1-87.5] | 66.7 [20.0-100.0] | 57.1 [25.0-84.2] | 61.5 [20.0-87.5] | 71.4 [41.3-100.0] | 76.1 [43.4-100.0] | 64.9 [29.1-92.7] | C=10, kernel=rbf, γ=scale |
| RandomForest | 72.2 [49.1-87.5] | 66.7 [20.0-100.0] | 57.1 [25.0-84.2] | 61.5 [22.2-88.9] | 72.1 [40.2-100.0] | 77.9 [43.6-100.0] | 65.3 [29.0-93.3] | n=20, depth=5, split=2 |
| LogisticRegression | 66.7 [43.7-83.7] | 55.6 [20.0-87.5] | 71.4 [35.9-91.8] | 62.5 [25.0-85.7] | 67.5 [37.6-97.5] | 65.6 [30.9-94.3] | 68.1 [33.1-92.5] | C=0.1, penalty=l2 |
| KNeighbors | 72.2 [49.1-87.5] | 66.7 [20.0-100.0] | 57.1 [25.0-84.2] | 61.5 [22.2-88.9] | 66.2 [35.0-97.5] | 68.1 [27.2-91.3] | 60.3 [25.6-87.9] | k=5, weights=uniform |
| DecisionTree | 72.2 [49.1-87.5] | 66.7 [20.0-100.0] | 57.1 [25.0-84.2] | 61.5 [20.0-87.5] | 71.4 [46.8-96.1] | 69.8 [25.0-88.9] | 60.9 [27.2-86.8] | depth=3, split=3 |
| GradientBoosting | 66.7 [43.7-83.7] | 57.1 [16.7-100.0] | 57.1 [25.0-84.2] | 57.1 [18.2-82.4] | 80.5 [59.7-100.0] | 73.6 [43.1-97.2] | 65.5 [33.1-90.0] | n=138, lr=0.056, depth=3 |
| AdaBoost | 72.2 [49.1-87.5] | 66.7 [20.0-100.0] | 57.1 [25.0-84.2] | 61.5 [22.2-88.9] | 70.1 [40.1-100.0] | 72.8 [41.1-95.8] | 64.0 [28.4-92.2] | n=52, lr=0.135 |
| GaussianNB | 83.3 [60.8-94.2] | 75.0 [40.0-100.0] | 85.7 [48.7-97.4] | 80.0 [50.0-100.0] | 83.1 [55.3-100.0] | 87.2 [59.7-100.0] | 84.4 [53.4-100.0] | Default |
| ExtraTrees | 72.2 [49.1-87.5] | 66.7 [20.0-100.0] | 57.1 [25.0-84.2] | 61.5 [22.2-88.9] | 64.3 [29.7-98.9] | 60.1 [29.2-96.7] | 60.0 [25.0-88.6] | depth=7, split=4 |
| MLP | 55.6 [33.7-75.4] | 42.9 [0.0-83.3] | 42.9 [15.8-75.0] | 42.9 [0.0-71.4] | 48.1 [11.0-85.1] | 61.6 [25.3-89.3] | 47.9 [6.6-80.6] | layers=(100,), α=0.0005 |

*Abbreviations: n = n_estimators, depth = max_depth, split = min_samples_split, lr = learning_rate, k = n_neighbors, α = alpha, γ = gamma. 95% CI computed using Wilson score (accuracy, recall) and bootstrap resampling (precision, F1, AUC, utility).*

**Table S4:** Classification results for the human dataset (n=22) using 10 machine learning models optimized via Grid Search, Genetic Algorithm (GA), and Particle Swarm Optimization (PSO). All metrics are presented as percentages with 95% confidence intervals in brackets. The utility score (0.4×recall + 0.2×F1 + 0.2×AUC-ROC + 0.2×AUC-PR) prioritizes recall for clinical screening. Gradient Boosting with PSO achieved the highest utility score (89.8%).

| **Grid Search** | | | | | | | | |
| --- | --- | --- | --- | --- | --- | --- | --- | --- |
| **Model** | **Accuracy** | **Precision** | **Recall** | **F1 Score** | **AUC-ROC** | **AUC-PR** | **Utility** | **Best Parameters** |
| SVC | 72.7 [51.8-86.8] | 63.6 [33.3-90.9] | 77.8 [45.3-93.7] | 70.0 [40.0-90.0] | 75.6 [49.3-100.0] | 81.1 [52.8-98.6] | 76.5 [49.5-95.7] | C=1, γ=scale, kernel=rbf |
| RandomForest | 86.4 [66.7-95.3] | 87.5 [60.0-100.0] | 77.8 [45.3-93.7] | 82.4 [55.6-100.0] | 88.9 [68.8-100.0] | 92.2 [74.7-100.0] | 83.8 [60.1-100.0] | depth=2, split=2, n=100 |
| LogisticRegression | 77.3 [56.6-89.9] | 75.0 [40.0-100.0] | 66.7 [35.4-87.9] | 70.6 [40.0-90.9] | 73.5 [48.0-99.0] | 77.6 [51.5-96.4] | 71.2 [41.9-94.9] | C=0.1, penalty=l2 |
| KNeighbors | 81.8 [61.5-92.7] | 77.8 [45.4-100.0] | 77.8 [45.3-93.7] | 77.8 [50.0-95.2] | 79.5 [54.0-100.0] | 85.4 [61.0-100.0] | 79.7 [53.2-98.7] | k=5, weights=distance |
| DecisionTree | 90.9 [72.2-97.5] | 88.9 [62.5-100.0] | 88.9 [56.5-98.0] | 88.9 [66.7-100.0] | 90.6 [77.4-100.0] | 91.2 [56.6-100.0] | 88.2 [68.0-100.0] | depth=3, split=2 |
| GradientBoosting | 90.9 [72.2-97.5] | 88.9 [62.5-100.0] | 88.9 [56.5-98.0] | 88.9 [66.7-100.0] | 82.9 [60.0-100.0] | 44.1 [57.1-100.0] | 86.7 [64.8-100.0] | lr=0.05, depth=3, n=100 |
| AdaBoost | 90.9 [72.2-97.5] | 88.9 [62.5-100.0] | 88.9 [56.5-98.0] | 88.9 [66.7-100.0] | 90.6 [77.4-100.0] | 91.2 [56.6-100.0] | 88.2 [68.0-100.0] | lr=0.1, n=50 |
| GaussianNB | 81.8 [61.5-92.7] | 77.8 [45.4-100.0] | 77.8 [45.3-93.7] | 77.8 [50.0-95.2] | 89.7 [73.8-100.0] | 91.1 [73.3-100.0] | 82.9 [60.1-98.9] | Default |
| ExtraTrees | 90.9 [72.2-97.5] | 100.0 [100.0-100.0] | 77.8 [45.3-93.7] | 87.5 [66.7-100.0] | 85.5 [64.3-100.0] | 89.0 [69.5-100.0] | 83.6 [59.1-100.0] | depth=5, split=2, n=100 |
| MLP | 68.2 [43.0-80.3] | 62.5 [25.0-100.0] | 55.6 [26.7-81.1] | 58.8 [25.0-83.3] | 81.2 [59.7-100.0] | 82.2 [58.4-98.0] | 66.8 [39.0-89.3] | α=0.0001, layers=(50,) |
| **Genetic Algorithm (GA)** | | | | | | | | |
| **Model** | **Accuracy** | **Precision** | **Recall** | **F1 Score** | **AUC-ROC** | **AUC-PR** | **Utility** | **Best Parameters** |
| SVC | 68.2 [43.0-80.3] | 62.5 [25.0-100.0] | 55.6 [26.7-81.1] | 58.8 [25.0-82.4] | 84.6 [66.8-100.0] | 85.4 [63.5-100.0] | 68.1 [43.0-87.7] | C=0.1, kernel=rbf, γ=scale |
| RandomForest | 86.4 [66.7-95.3] | 87.5 [60.0-100.0] | 77.8 [45.3-93.7] | 82.4 [55.6-100.0] | 89.7 [71.2-100.0] | 92.4 [75.6-100.0] | 84.1 [61.2-100.0] | n=20, depth=5, split=2 |
| LogisticRegression | 77.3 [56.6-89.9] | 75.0 [40.0-100.0] | 66.7 [35.4-87.9] | 70.6 [40.0-90.9] | 69.2 [38.7-99.8] | 79.4 [53.8-100.0] | 70.6 [39.3-96.0] | C=1, penalty=l2 |
| KNeighbors | 81.8 [61.5-92.7] | 77.8 [45.5-100.0] | 77.8 [45.3-93.7] | 77.8 [50.0-95.2] | 72.2 [46.5-97.9] | 67.7 [35.8-95.5] | 74.0 [47.9-95.2] | k=5, weights=uniform |
| DecisionTree | 90.9 [72.2-97.5] | 88.9 [62.5-100.0] | 88.9 [56.5-98.0] | 88.9 [66.7-100.0] | 90.6 [77.4-100.0] | 91.2 [56.6-100.0] | 88.2 [68.0-100.0] | depth=3, split=2 |
| GradientBoosting | 90.9 [72.2-97.5] | 88.9 [62.5-100.0] | 88.9 [56.5-98.0] | 88.9 [66.7-100.0] | 88.0 [71.4-100.0] | 82.0 [59.2-100.0] | 87.9 [67.7-100.0] | n=100, lr=0.1, depth=5 |
| AdaBoost | 90.9 [72.2-97.5] | 88.9 [62.5-100.0] | 88.9 [56.5-98.0] | 88.9 [66.7-100.0] | 90.6 [77.4-100.0] | 91.2 [56.6-100.0] | 88.2 [68.0-100.0] | n=100, lr=0.1 |
| GaussianNB | 81.8 [61.5-92.7] | 77.8 [45.4-100.0] | 77.8 [45.3-93.7] | 77.8 [50.0-95.2] | 89.7 [73.8-100.0] | 91.1 [73.3-100.0] | 82.9 [60.1-98.9] | Default |
| ExtraTrees | 90.9 [72.2-97.5] | 100.0 [100.0-100.0] | 77.8 [45.3-93.7] | 87.5 [66.7-100.0] | 85.5 [64.3-100.0] | 89.0 [69.5-100.0] | 83.6 [59.1-100.0] | n=100, depth=5, split=4 |
| MLP | 54.5 [34.7-73.1] | 46.7 [21.4-73.3] | 77.8 [45.3-93.7] | 58.3 [30.0-80.0] | 59.0 [34.0-84.0] | 51.3 [25.1-78.2] | 63.8 [41.0-83.9] | layers=(20,), α=0.0001 |
| **Particle Swarm Optimization (PSO)** | | | | | | | | |
| **Model** | **Accuracy** | **Precision** | **Recall** | **F1 Score** | **AUC-ROC** | **AUC-PR** | **Utility** | **Best Parameters** |
| SVC | 77.3 [56.6-89.9] | 70.0 [37.5-100.0] | 77.8 [45.3-93.7] | 73.7 [44.4-92.3] | 79.5 [55.4-100.0] | 83.2 [59.1-99.2] | 78.5 [52.4-97.0] | C=10, kernel=rbf, γ=scale |
| RandomForest | 90.9 [72.2-97.5] | 88.9 [62.5-100.0] | 88.9 [56.5-98.0] | 88.9 [66.7-100.0] | 88.9 [68.8-100.0] | 92.2 [74.7-100.0] | 89.6 [68.1-100.0] | n=20, depth=5, split=2 |
| LogisticRegression | 81.8 [61.5-92.7] | 85.7 [50.0-100.0] | 66.7 [35.4-87.9] | 75.0 [42.9-94.7] | 76.1 [53.4-98.8] | 76.1 [49.5-96.5] | 72.3 [44.0-95.1] | C=1, penalty=l1 |
| KNeighbors | 86.4 [66.7-95.3] | 87.5 [60.0-100.0] | 77.8 [45.3-93.7] | 82.4 [55.6-100.0] | 78.6 [53.3-100.0] | 83.7 [59.0-100.0] | 80.1 [53.9-100.0] | k=5, weights=distance |
| DecisionTree | 90.9 [72.2-97.5] | 88.9 [62.5-100.0] | 88.9 [56.5-98.0] | 88.9 [66.7-100.0] | 90.6 [77.4-100.0] | 91.2 [56.6-100.0] | 88.2 [68.0-100.0] | depth=5, split=3 |
| **GradientBoosting** | **90.9 [72.2-97.5]** | **88.9 [62.5-100.0]** | **88.9 [56.5-98.0]** | **88.9 [66.7-100.0]** | **91.5 [78.3-100.0]** | **90.5 [70.9-100.0]** | **89.8 [70.1-100.0]** | **n=130, lr=0.053, depth=5** |
| AdaBoost | 90.9 [72.2-97.5] | 88.9 [62.5-100.0] | 88.9 [56.5-98.0] | 88.9 [66.7-100.0] | 90.6 [77.4-100.0] | 91.2 [56.6-100.0] | 88.2 [68.0-100.0] | n=60, lr=0.528 |
| GaussianNB | 81.8 [61.5-92.7] | 77.8 [45.4-100.0] | 77.8 [45.3-93.7] | 77.8 [50.0-95.2] | 89.7 [73.8-100.0] | 91.1 [73.3-100.0] | 82.9 [60.1-98.9] | Default |
| ExtraTrees | 86.4 [66.7-95.3] | 87.5 [60.0-100.0] | 77.8 [45.3-93.7] | 82.4 [55.6-100.0] | 85.5 [64.3-100.0] | 89.0 [69.5-100.0] | 82.6 [58.0-100.0] | depth=8, split=3 |
| MLP | 81.8 [61.5-92.7] | 77.8 [50.0-100.0] | 77.8 [45.3-93.7] | 77.8 [50.0-95.2] | 86.3 [68.3-100.0] | 87.5 [66.4-100.0] | 81.5 [56.9-98.2] | layers=(50,), α=0.0005 |

*Abbreviations: n = n_estimators, depth = max_depth, split = min_samples_split, lr = learning_rate, k = n_neighbors, α = alpha, γ = gamma. 95% CI computed using Wilson score (accuracy, recall) and bootstrap resampling (precision, F1, AUC, utility).*

**Table S5:** Selected features (n=32) for classification model. Features were identified through multi-stage selection: univariate filtering (Mann-Whitney U test, p<0.05; mutual information >0.02), multivariate analysis (MANOVA, LDA, pairwise AUC ≥0.85), and correlation pruning (Pearson r<0.90). Statistical features computed over entire time series; temporal features capture dynamics around maximum grayscale transition; delta features quantify color change magnitude.

| **#** | **Feature** | **Color Channel** | **Type** | **Description** |
| --- | --- | --- | --- | --- |
| 1 | B_max | Blue (B) | Statistical | Maximum value |
| 2 | B_mean | Blue (B) | Statistical | Mean value |
| 3 | B_min | Blue (B) | Statistical | Minimum value |
| 4 | B_post3_mean | Blue (B) | Temporal | Mean of 3 frames post-transition |
| 5 | B_pre3_mean | Blue (B) | Temporal | Mean of 3 frames pre-transition |
| 6 | G_max | Green (G) | Statistical | Maximum value |
| 7 | G_mean | Green (G) | Statistical | Mean value |
| 8 | G_min | Green (G) | Statistical | Minimum value |
| 9 | G_post3_mean | Green (G) | Temporal | Mean of 3 frames post-transition |
| 10 | G_pre3_mean | Green (G) | Temporal | Mean of 3 frames pre-transition |
| 11 | G_std | Green (G) | Statistical | Standard deviation |
| 12 | Gray_Rec601_max | Grayscale (Rec. 601) | Statistical | Maximum value |
| 13 | Gray_Rec601_mean | Grayscale (Rec. 601) | Statistical | Mean value |
| 14 | Gray_Rec601_min | Grayscale (Rec. 601) | Statistical | Minimum value |
| 15 | Gray_Rec601_post3_mean | Grayscale (Rec. 601) | Temporal | Mean of 3 frames post-transition |
| 16 | Gray_Rec601_pre3_mean | Grayscale (Rec. 601) | Temporal | Mean of 3 frames pre-transition |
| 17 | Gray_Rec601_std | Grayscale (Rec. 601) | Statistical | Standard deviation |
| 18 | H_mean | Hue (H) | Statistical | Mean value |
| 19 | L_lab_min | CIE Lab L* | Statistical | Minimum value |
| 20 | L_lab_std | CIE Lab L* | Statistical | Standard deviation |
| 21 | R_max | Red (R) | Statistical | Maximum value |
| 22 | R_mean | Red (R) | Statistical | Mean value |
| 23 | R_min | Red (R) | Statistical | Minimum value |
| 24 | R_post3_mean | Red (R) | Temporal | Mean of 3 frames post-transition |
| 25 | R_std | Red (R) | Statistical | Standard deviation |
| 26 | S_mean | Saturation (S) | Statistical | Mean value |
| 27 | S_min | Saturation (S) | Statistical | Minimum value |
| 28 | V_max | Value (V) | Statistical | Maximum value |
| 29 | V_mean | Value (V) | Statistical | Mean value |
| 30 | delta_G | Green (G) | Delta | Post-transition minus pre-transition mean |
| 31 | delta_Gray_Rec601 | Grayscale (Rec. 601) | Delta | Post-transition minus pre-transition mean |
| 32 | delta_R | Red (R) | Delta | Post-transition minus pre-transition mean |

*Color spaces: RGB (Red, Green, Blue), HSV (Hue, Saturation, Value), CIE Lab (L* lightness channel), Grayscale (Rec. 601 luminance: 0.299R + 0.587G + 0.114B).*

**Table S6:** Blind test classification performance with 95% confidence intervals.

| **Metric** | **Value (%)** | **95% CI (%)** | **Method** |
| --- | --- | --- | --- |
| Accuracy | 90.0 | 59.6–98.2 | Wilson |
| Precision | 80.0 | 40.0–100.0 | Bootstrap |
| Recall | 100.0 | 51.0–100.0 | Wilson |
| Specificity | 83.3 | 43.6–97.0 | Wilson |
| F1 score | 88.9 | 57.1–100.0 | Bootstrap |
| AUC-ROC | 91.7 | 75.3–100.0 | DeLong |
| AUC-PR | 90.0 | 70.0–100.0 | Bootstrap |
| Utility score | 92.1 | 74.9–100.0 | Bootstrap |

Table S7: Cost estimates of our assay

| **Component** | **Typical Amount per Test** | **Estimated Cost per Test (USD)** |
| --- | --- | --- |
| Streptavidin-coated magnetic particles | ~100 µg | ~$0.20 |
| Biotinylated cTnI aptamer | ~100 pmol | ~$0.05 |
| Complementary locking strand | ~100 pmol | ~$0.05 |
| HCR hairpins (HTG-H1 to HTG-H4) | 1–2 µM each | ~$0.40 |
| Hemin (DNAzyme cofactor) | ~1 µM | ~$0.05 |
| ABTS chromogenic substrate | ~1–2 mM | ~$0.05 |
| Hydrogen peroxide | Trace | <$0.01 |
| Blocking reagents (BSA, Tween-20) | — | ~$0.05 |
| Buffers and salts | — | ~$0.05 |
| **3D-printed disposable cartridge** | Single-use | ~$0.40 |
| **Webcam-based optical readout (amortized)** | Reusable | ~$0.60 |
| **Estimated total cost per test** | — | **~$1.9–2.0** |

Reference:

[1] “Amplified colorimetric detection of tetracycline based on an enzyme-linked aptamer assay with multivalent HRP-mimicking DNAzyme - Analyst (RSC Publishing),” can be found under https://pubs.rsc.org/en/content/articlelanding/2019/an/c8an02284d, **n.d.**
